# Initial State Encoding via Reverse Quantum Annealing and H-Gain Features

**DOI:** 10.1109/tqe.2023.3319586

**Published:** 2023-09-27

**Authors:** ELIJAH PELOFSKE, GEORG HAHN, HRISTO DJIDJEV

**Affiliations:** 1 CCS-3 Information Sciences, Los Alamos National Laboratory, Los Alamos, NM 87545 USA; 2 Harvard T. H. Chan School of Public Health, Harvard University, Boston, MA 02115 USA; 3 Institute of Information and Communication Technologies, Bulgarian Academy of Sciences, 1040 Sofia, Bulgaria

**Keywords:** Anneal schedule, Bayesian optimization, D-Wave, h-gain (HG), Ising model, maximum cut, QUBO, quantum annealing (QA), reverse annealing (RA)

## Abstract

Quantum annealing is a specialized type of quantum computation that aims to use quantum fluctuations in order to obtain global minimum solutions of combinatorial optimization problems. Programmable D-Wave quantum annealers are available as cloud computing resources, which allow users low-level access to quantum annealing control features. In this article, we are interested in improving the quality of the solutions returned by a quantum annealer by encoding an initial state into the annealing process. We explore twoD-Wave features that allow one toencode such an initialstate: the reverse annealing (RA) and theh-gain(HG)features.RAaimstorefineaknownsolutionfollowinganannealpathstartingwithaclassical state representing a good solution, going backward to a point where a transverse field is present, and then finishing the annealing process with a forward anneal. The HG feature allows one to put a time-dependent weighting scheme on linear (h) biases of the Hamiltonian, and we demonstrate that this feature likewise can be used to bias the annealing to start from an initial state. We also consider a hybrid method consisting of a backward phase resembling RA and a forward phase using the HG initial state encoding. Importantly, we investigate the idea of iteratively applying RA and HG to a problem, with the goal of monotonically improving on an initial state that is not optimal. The HG encoding technique is evaluated on a variety of input problems including the edge-weighted maximum cut problem and the vertex-weighted maximum clique problem, demonstrating that the HG technique is a viable alternative to RA for some problems. We also investigate how the iterative procedures perform for both RA and HG initial state encodings on random whole-chip spin glasses with the native hardware connectivity of the D-Wave Chimera and Pegasus chips.

## I. INTRODUCTION

Quantum annealing (QA) is a form of specialized quantum computation that uses quantum fluctuations in order to search for the global minimum of a combinatorial optimization problem [1], [2], [3], [4], [5], [6]. Programmable quantum annealers, available as cloud computing resources, are manufactured by D-Wave Systems, Inc., using superconducting flux qubits [7], [8], [9], [10], [11], [12]. Quantum annealers are designed to minimize quadratic forms (called Hamiltonian functions), defined by

(1) $$Q\left(x_{1},\ldots ,x_{n}\right)=\sum_{i=1}^{n}h_{i}x_{i}+\sum_{i<j}J_{ij}x_{i}x_{j}.$$

The variables $x_{i}$ in (1) are unknown and take binary values only. The coefficients $h_{i}\in \mathbb{R}$ (linear weights) and $J_{ij}\in \mathbb{R}$ (quadratic couplers) are chosen by the user to define the problem under investigation, where i, j∈{1,…,n}. If the variables $x_{i}\in \{0,1\}$, then (1) is called a *quadratic unconstrained binary optimization* (QUBO) problem, and if $x_{i}\in \{-1,+1\}$, it is called an *Ising problem*, and importantly, QUBO and Ising formulations are equivalent. Many important NP-hard problems map to simple minimization problems of the type of (1) (see [13]).

With newer generations of the D-Wave quantum annealer, an increasing number of features have been added to the machines that allow the user to obtain greater control over the annealing process. Such features include the spin reversal transform, customized anneal schedules, or anneal offsets for individual qubits. In this contribution, we focus on two of the latest features, called reverse annealing (RA) and *time-dependent gain in linear biases*, also known as *h-gain* (HG). Both RA and HG protocols are specified as time-dependent schedules over the course of the annealing process.

This article investigates how both the RA and HG features can be used to improve the quality of an initial (suboptimal) solution, which is used to seed the annealing process. In fact, the purpose of RA is to guide the annealing process from a known classical (suboptimal) state to a point where more of the transverse field Hamiltonian is present, before reversing direction again and continuing with a standard forward anneal (FA) (optionally with more complicated schedules, or simply to pause at an anneal fraction for some duration). We show that the HG feature allows one to achieve a similar goal as RA, though through a different mechanism. For both the methods, one hopes that seeding the anneal with a solution that is close to optimal will help the annealer to transition to a better minimum, thereby improving the best found solution.

The HG feature allows one to put an additional weight on the linear terms in (1) in a time-dependent way. The feature was introduced to better study freeze-out points [7] and phase transitions in spin glasses [14]. We show in this work that HG can also be used to bias the annealing process toward an initially computed solution. However, in contrast to RA, we only use an FA. The mechanism we employ works as follows. We assume an Ising formulation in (1) with no linear terms. We then add to the given Ising formulation a new linear term that serves as a bias toward the known initial solution. Using the HG feature, we can put maximal weight on the linear terms at the start of the annealing process that biases the anneal toward the initial state and decrease the HG strength during the annealing process to zero in order to make the annealer explore nearby solutions. An extension of this idea to Ising formulations with linear terms is presented as well. Finally, we explore schedules that combine both RA and HG in that they use a backward phase resembling an RA step and a forward phase that uses our HG idea.

Both the RA and HG techniques investigated in this work have a variety of tunable parameters. In particular, the RA feature requires the specification of annealing time duration and anneal schedule. The HG feature likewise requires annealing time, HG schedule governing the time-dependent linear biases, and up to two additional parameters used to scale the appended linear terms in relation to the existing terms in (1). To tune those parameters, we employ a Bayesian optimization framework [15]. The details of all optimizations being performed are given in this article, together with the best anneal schedules we found for RA and HG to encode initial solutions. These insights may prove valuable for users programming RA and HG schedules on D-Wave quantum annealers.

This article is a journal version and substantial extension of the conference paper of [16], published in the *2020 IEEE International Conference on Quantum Computing and Engineering*. In addition to the conference version, the present journal article also considers an application of RA and HG to Quantum Evolution Monte Carlo (QEMC), also known as iterative RA. We demonstrate that both RA and HG can be employed in QA to encode initial states in each iteration. We investigate the performance of such QEMC algorithms on random spin glasses with native D-Wave connectivity across three different D-Wave quantum processing units (QPUs). The evaluated D-Wave QPUs have native hardware graphs known as Chimera (specifically with size $C_{16}$) [17] and Pegasus (specifically with size $P_{16}$) [17], [18], [19].

The rest of this article is organized as follows. We start with a brief literature review (see Section II) before introducing details on how to encode an initial state prior to an annealing process using the RA and/or HG features (see Section III). That section also describes how we employ RA and HG in connection with QEMC. Section IV presents experimental results for the edge-weighted maximum cut problem (see Section IV-B), the vertex-weighted maximum clique problem (see Section IV-C), as well as QEMC (see Section IV-D). Finally, Section V concludes this article. Certain tuning parameters we employed in our experiments are given in the Appendixes.

## II. PREVIOUS WORK

The new HG feature has not received too much attention in the literature to date. However, RA has been studied by several authors. Its idea was first introduced in [20] under the name of *sombrero adiabatic quantum computation*. Using tests on 3-SAT instances, the authors noted that the performance of RA was dependent on the Hamming distance between the planted initial solution and the optimal solution. However, the methodology of [20] differs from the dissipative protocol actually used on the D-Wave devices in their RA feature. The latter is closer to the protocol of Chancellor [21], who demonstrates how sequential calls to quantum annealers can be used to construct analogues of population annealing and parallel tempering which use quantum searches as subroutines.

The term “RA” is not uniquely defined in the literature and might refer to distinct protocols. An overview of those protocols can be found in [22].

Since its introduction, RA has been used in a wide range of practical applications, ranging from matrix factorization [23] to first attempts on neural networks [24] and portfolio optimization [25].

A theoretical contribution can be found in [26], where the authors study conditions under which RA can lead to improvements over standard annealing for the fully connected p spin model. They present a theoretical framework to characterize such cases, but remark that their results do not necessarily apply to experimental setups where RA is performed adiabatically and in a thermal environment.

An application of adiabatic RA, which is an FA similar to the HG version studied here, and iterative RA as used in the D-Wave annealer, is analyzed in [27], using direct numerical integration of the time-dependent Schrödinger equation. The authors find a theoretical speedup of adiabatic RA over QA for mean-field-type p-spin models. However, they also find that iterative RA as used by D-Wave does not provide such advantage in theory, which is attributed to the fact that D-Wave is not a closed system, meaning that the theoretical results may not apply. However, a later publication [28] concludes that, indeed, standard QA can outperform adiabatic RA with decoherence.

A considerable speedup of RA over QA in a real-world application (portfolio optimization) is reported in [29], under the condition that RA starts from a planted heuristic solution.

Some differences of open-system dynamics versus closed-system RA is studied empirically in [30] using three-spin models. The authors observe that RA with a pause converges to the ground state with a higher success probability than purely closed-system RA, prompting the authors to conjecture that the open-system dynamics makes RA work in devices such as the D-Wave annealer.

An interesting recent development in the field pertains to biased search protocols. Those work by initializing the anneal in an unequal (inhomogeneous) superposition of the possible states, thereby biasing the annealing dynamics into the desired solution using longitudinal fields [31], [32], which is a very similar idea to the D-Wave hardware HG field (except that the HG field is uniformly applied to all qubits).

## III. METHODS

This section describes the techniques we use to encode an initial solution, both via the D-Wave’s RA feature (see Section III-A) and with the help of a suitably chosen additional linear term in connection with the HG feature (see Sections III-B and III-C). Section III-D describes the combined technique of RA+HG. Section III-E states the final Hamiltonian we optimize and briefly describes the Bayesian optimization framework used to optimize the reverse QA and HG schedules. Section III-F defines the iterative state encoding procedure, also known as QEMC, when applied using reverse QA and the HG initial state encoding method.

### A. ANNEAL PATHS BASED ON RA

**FIGURE 1.**
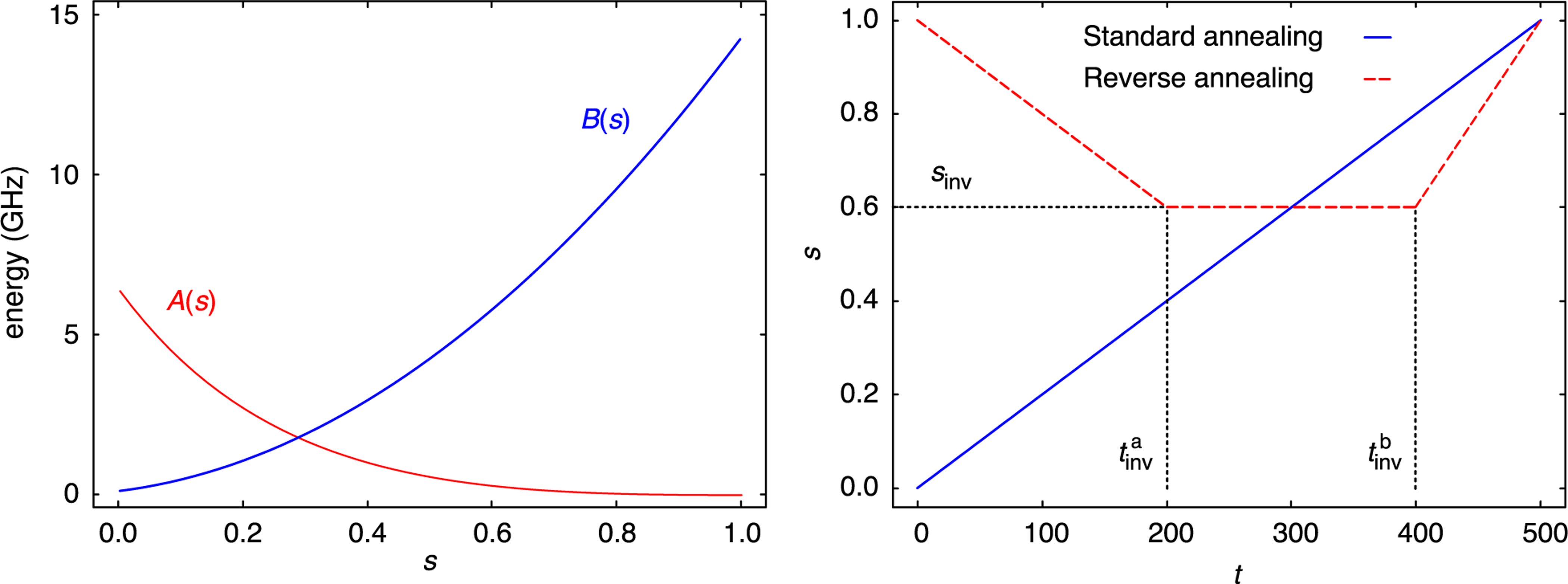
(Left) Functions A(s) and B(s) controlling the annealing process, where s∈[0,1] is the anneal fraction. (Right) Progression of the anneal fraction s for standard forward and RA with pause as a function of time t∈[0,500]μs. Figure adapted from [30]. Note that the functions of A(s) and B(s) will change slightly depending on the quantum annealer.

In a standard FA, all qubits are prepared in an equal super-position of all states, as determined by the transverse field portion of the system’s Hamiltonian. During annealing, the amplitude of the transverse field is being decreased toward 0, while the Hamiltonian is slowly transformed into a Hamiltonian corresponding to the Ising problem being minimized. Specifically, the evolution of D-Wave’s quantum system is described by the following time-dependent Hamiltonian:

(2) $$H(s)=-\frac{A(s)}{2}\left(\sum_{i=1}^{n}{\hat{\sigma }}_{x}^{\left(i\right)}\right)\;\;\;\;+\frac{B(s)}{2}\left(\sum_{i=1}^{n}h_{i}{\hat{\sigma }}_{z}^{\left(i\right)}+\sum_{i\leq j}J_{ij}{\hat{\sigma }}_{z}^{\left(i\right)}{\hat{\sigma }}_{z}^{\left(j\right)}\right)$$

where the first term having the prefactor −A(s)/2 is the transverse field and the term following the prefactor B(s)/2 is the Hamiltonian corresponding to the Ising model of (1), and ${\hat{\sigma }}_{x}^{\left(i\right)}$ and ${\hat{\sigma }}_{z}^{\left(i\right)}$ are the Pauli x and z operators operating on qubit i. The specific functions A(s) and B(s) used for the D-Wave 2000Q machine at Los Alamos are shown in Fig. 1(left). These functions are indexed by a parameter s∈[0,1] called the *anneal fraction*, which itself is a function s(t) of the time t. In the case of the FA, it is given as s(t)=t/T, where T is the total annealing time.

In contrast to FA, RA starts with a precomputed classical solution that is expected to be much closer in quality to an optimal one than a random starting point. Then, a two-stage process is initiated (see the red curve in Fig. 1, right), during which quantum fluctuations are first increased by reducing the anneal fraction from s=1 to a value $s_{\text{inv}}\in (0,1)$ at time $t_{\text{inv}}^{a}$. After the turning point is reached, and after an optional pause until time $t_{\text{inv}}^{b}$, the anneal follows again the path of a standard FA from $s_{\text{inv}}$ up to s=1 at full annealing time T. Careful choices of the turning point and the initial state can lead to improvements in the solution compared to an FA [20], [33].

### B. ANNEAL PATHS BASED ON THE HG SCHEDULE

The feature of a *time-dependent gain in Hamiltonian linear biases* allows the user to have more control of the annealing process by biasing linear terms of an Ising model with the help of a time-dependent function g(t) as follows (see [14]):

(3) $$H_{\text{HG}}(s)=-\frac{A(s)}{2}\left(\sum_{i=1}^{n}{\hat{\sigma }}_{x}^{\left(i\right)}\right)\;\;\;\;+\frac{B(s)}{2}\left(\sum_{i=1}^{n}g(t)h_{i}{\hat{\sigma }}_{z}^{\left(i\right)}+\sum_{i>j}J_{ij}{\hat{\sigma }}_{z}^{\left(i\right)}{\hat{\sigma }}_{z}^{\left(j\right)}\right)$$

Compared to (2), we see that the linear terms of the Ising model in (3) are weighted with a function g(t), specified by the user, which controls the time-dependent gain for the linear terms. In our implementation, we initialize the function with g(0)∈[0,5] (5 being the largest value allowed for D-Wave 2000Q) and decrease it to g(T)=0 using up to 20 points on the schedule. The specification of the HG feature is actually more general than the way we use it in this work. For instance, the function g(t) may actually return values in [−5,5], it does not need to be monotonic, and there are a (machine-dependent) upper bound of 500 for the slope between changes in the schedule and a (machine-dependent) upper bound of 20 on the number of points determining the schedule [34].

Our aim is to employ the HG feature to encode an initial solution at the start of the annealing process. Assume that we are given an Ising problem of the type of (1) with no linear term, i.e., $h_{i}=0$ for all i. The idea lies in the observation that, for a fixed initial value $x^{\left(0\right)}=\left(x_{1}^{0},\ldots ,x_{n}^{0}\right)\in {\left\{-1,+1\right\}}^{n}$, the minimum of the special Ising function containing only linear terms

(4) $$h(x)=\sum_{i=1}^{n}\left(-x_{i}^{0}\right)x_{i}$$

for $x=\left(x_{1},\ldots ,x_{n}\right)$, is equal to −n, and it occurs at $x=x^{\left(0\right)}$. Hence, we can define $h_{i}=-x_{i}^{0}$ for i=1,…,n and use an HG anneal schedule of the type of (3). By putting a large weight on the linear terms at the start of the anneal using the function g(t), we bias the annealing solution toward our planted solution $x^{\left(0\right)}$. Over the course of the anneal, the HG bias [the function g(t) in (3)] is decreased toward 0, thus allowing the annealing process to move away from the planted solution and to explore alternative solutions in its neighborhood.

However, in order for this idea to work, the original Ising model may not have a linear term, so we can create our own linear term to encode the initial solution. For instance, maximum cut, graph partitioning, and number partitioning are such NP-hard problems without linear terms [13]. Most Ising formulations of NP-hard problems, however, seem to have linear terms. Next, we will show that, even for such problems, the HG approach can be applicable.

### C. USING HG FOR ISING PROBLEMS CONTAININGLINEAR TERMS

For problems whose Ising formulations do have linear terms, we apply the following transformation to eliminate them. First, we homogenize the polynomial in (1) by converting the linear term into a quadratic one. This is achieved by introducing a new variable z∈{−1,+1}, which we call a *slack variable*. The slack variable z is multiplied with each linear term, thus transforming (1) into

(5) $$Q^{\prime }(x,z)=\sum_{i=1}^{n}h_{i}x_{i}z+\sum_{i<j}J_{ij}x_{i}x_{j}.$$

Note that Q can be recovered from $Q^{\prime }$ by setting z=1. Now, we can apply the method, as discussed in Section III-B. After the end of the annealing process, we ignore all solutions with z=−1. We can guide the annealing process to favor solutions with z=1 by using an appropriate HG bias (initial solution).

### D. RA AND HG COMBINED

The ideas of RA and HG can actually be combined into a single D-Wave schedule. To be precise, given an initial solution $x^{\left(0\right)}$ to be encoded, we first apply the methodology of Sections III-B and III-C to arrive at a new Ising model encoding $x^{\left(0\right)}$. We then solve the new Ising model using an RA schedule, which specifies the anneal fraction s as a function of time, combined with an HG schedule, which specifies the gain g(t) as a function of time.

If the HG Hamiltonian computed in Sections III-B and III-C requires a slack variable z, we also need to supply an initial state for z when running RA. In order to reinforce z=1, we simply set z=1 in the RA initial state additionally to $x=x^{\left(0\right)}$. Note that the two schedules for RA+HG contain a total number of five parameters: three parameters for the RA schedule and two parameters for the HG schedule.

### E. FINAL HAMILTONIAN AND TUNING OF PARAMETERS FOR MINOR EMBEDDED COMBINATORIAL OPTIMIZATION PROBLEM INSTANCES

To arrive at an effective implementation of the RA and HG methods, we need to determine appropriate values for a set of parameters, some optional, others required.

For HG, optional parameters are the coefficients $h_{i}$ from (3), for which we have so far suggested only their sign in (4). While choosing individual weights for each $h_{i}$ will result in highest accuracy, it is also the most difficult to accomplish and beyond the scope of this article. Instead, we use a single coefficient $\alpha_{1}$ for i=1,…,n and, in the case when we need to homogenize the input Ising model, another coefficient $\alpha_{2}$ for the new variable z.

Combining the above, we encode an initial state using the Ising model

(6) $$Q_{\text{final}}(x,z)=\alpha_{1}\left(\sum_{i=1}^{n}\left(-x_{i}^{0}\right)x_{i}\right)-\alpha_{2}z+Q^{\prime }(x,z)$$

which is a function of $x_{1},\ldots ,x_{n}$ and z. The two scaling constants $\alpha_{1}$ and $\alpha_{2}$ allow us to control the strength enforcing the bias toward the initial solution and the condition that z=1. If the Ising model under consideration in (1) does not have a linear term, no new variable z is needed, and thus, $\alpha_{2}=0$ in (6). When implementing (6) on the D-Wave QPU, we set the internal option autoscale to on (which is the default option), thus scaling all quadratic terms to the range [−1,+1] when programmed on DW_2000Q_LANL.

Apart from $\alpha_{1}$ and $\alpha_{2}$ in (6), the parameters that are required for both RA and HG are the schedule parameters. For RA, we specifically target RA schedules with a single pause. Therefore, for RA, we need the values $t_{\text{inv}}^{a}$, $t_{\text{inv}}^{b}$, and $s_{\text{inv}}$ (see Fig. 1) plus the total annealing time T. For HG, we need the function g(t) given as a polygonal line subject to D-Wave’s restrictions on magnitude, angles, and number of points. While there is some previous work that can be used as a guide for setting the schedule, in the case of HG, there is no such previous work. Hence, we apply an optimization procedure for choosing the HG parameters, and in order to make a fair comparison between RA and HG, we use the same method for choosing the RA parameters.

We employ the following Bayesian optimization procedure to tune the aforementioned parameters for the minor embedded combinatorial optimization problems, all on the D-Wave QA device with chip id DW_2000Q_LANL. The tuning is done separately for the two classes of minor embedded problems (weighted maximum clique and weighted maximum cut) that we study in more detail in the experiments of Section IV, i.e., the maximum cut and maximum clique problems, as follows.

1. We first fix the anneal time T and then the anneal schedule for RA. After having determined T, we fix the starting point (t=0, s=1) and the end point (t=T, s=1) [see Fig. 1(right)]. As in Fig. 1(right), we decrease the anneal fraction s to a point ($t_{\text{inv}}^{a}$, $s_{\text{inv}}$). We then allow for a pause, meaning that we also allow a point ($t_{\text{inv}}^{b}$, $s_{\text{inv}}$) at the same $s_{\text{inv}}$. All in all, we need to determine four parameters for RA: T, $t_{\text{inv}}^{a}$, $t_{\text{inv}}^{b}$, and $s_{\text{inv}}$. In practice, the way that the Bayesian optimization procedure for RA (with a pause) is parameterized is using two real numbers both in range [0.1,0.9]—the first real number specifies at what point in the anneal (as a proportion of the total annealing), and the second number specifies the pause duration (as a proportion out of the available annealing time after the pause begins). These two parameters can completely specify symmetric RA schedules with pauses. Together, the extreme ranges for these two parameters could allow the Bayesian optimizer to propose schedules, which would cause the D-Wave quantum annealer to return an error due to the anneal schedule changing too quickly, assuming that the full range of annealing times could be applied. Therefore, for RA schedule Bayesian optimization experiments, we set the minimum allowed annealing time to be 100 *μ*s; this ensures that all schedules that the Bayesian optimizer would propose are valid. Specifically, this constraint means that the extreme choices of the RA schedule parameters could result in a change from s=0 to s=1 in 1 *μ*s, which is within the hardware anneal schedule slope constraints for DW_2000Q_LANL.
2. Similarly, for the HG schedule, we first fix T and then the schedule’s end points, starting at (0,5) and ending at (T,0). We allow for one point in-between, (h, t), where h∈[0,5] and t∈(0,1). Together, three parameters are required for HG, that is, T, h, and t. Note that such a shape for an HG schedule is by no means optimal, but we want to keep the number of parameters low to have a more manageable search space. However, before determining the schedule parameters, we first determine the best scaling factors $\alpha_{1}$ and $\alpha_{2}$ in (6). If the Ising model under consideration in (1) only has quadratic terms, homogenizing the polynomial is not necessary, and we thus only need to find $\alpha_{1}$ in the Hamiltonian of (6). Otherwise, both $\alpha_{1}$ and $\alpha_{2}$ are determined. In practice, on the hardware, there are limitations on the slope of the HG schedule. These maximum HG schedule slopes are device dependent, but on the quantum annealer that was used for the minor embedded combinatorial optimization problems, which is DW_2000Q_LANL, we constrained the Bayesian optimization search space for the HG point during the anneal to be between [0.01,0.99] as a proportion out of the total annealing time. Without these constraints, it would be possible for the Bayesian optimizer to propose schedules, which would cause the D-Wave back end to return an error. Under this constraint, the smallest annealing time available on DW_2000Q_LANL, 1 *μ*s, is feasible for the Bayesian optimization to be applied since this would cause at most an HG field strength change of 0.01 *μ*s, which was within the HG field parameters of the device.
3. For the combined technique of RA+HG, after having determined the scaling constants $\alpha_{1}$ and $\alpha_{2}$ and the total annealing time T, we are left with five parameters determining the schedules: $t_{\text{inv}}^{a}$, $t_{\text{inv}}^{b}$, and $s_{\text{inv}}$ for RA, and h and t for HG.

To optimize all these parameters, we employ the Bayesian optimization tool of [35]. Bayesian optimization [15], [36], [37] is a sequential optimization strategy to find the global optimum of a smooth function without the need for derivatives. An advantage of Bayesian optimization and the reason we chose it in this research is the fact that it also works with functions that are noisy, which is the case here since the function we optimize is based on the energy values returned by the D-Wave quantum annealer.

### F. QUANTUM EVOLUTION MONTE CARLO

A straightforward extension of the methodology presented in Sections III-A and III-B is to iteratively apply the initial state encoding over some number of iterations, where the best solution from each previous iteration serves as the seed of the new anneal. This idea often referred to in the literature as QEMC [33], [38], [39], [40], [41] also referred to as *iterated RA* [27], [42], [43]. Initial state encodings are also known as warm starting in other contexts [44], [45], [46], [47]. Such approaches have been used for a variety of physics simulation computations on quantum annealers. By iteratively seeding each new anneal with the previously (best) obtained solution, one hopes to incrementally improve upon the solution quality. In the rest of this article, we will refer to this method as QEMC.

We aim to employ both RA and HG to encode the initial states in QEMC before each new iteration. As in Section III-E, these schedules have a variety of tuning parameters that we need to determine. Specifically, we test the following four different iterated state encoding methods.

**FIGURE 2.**
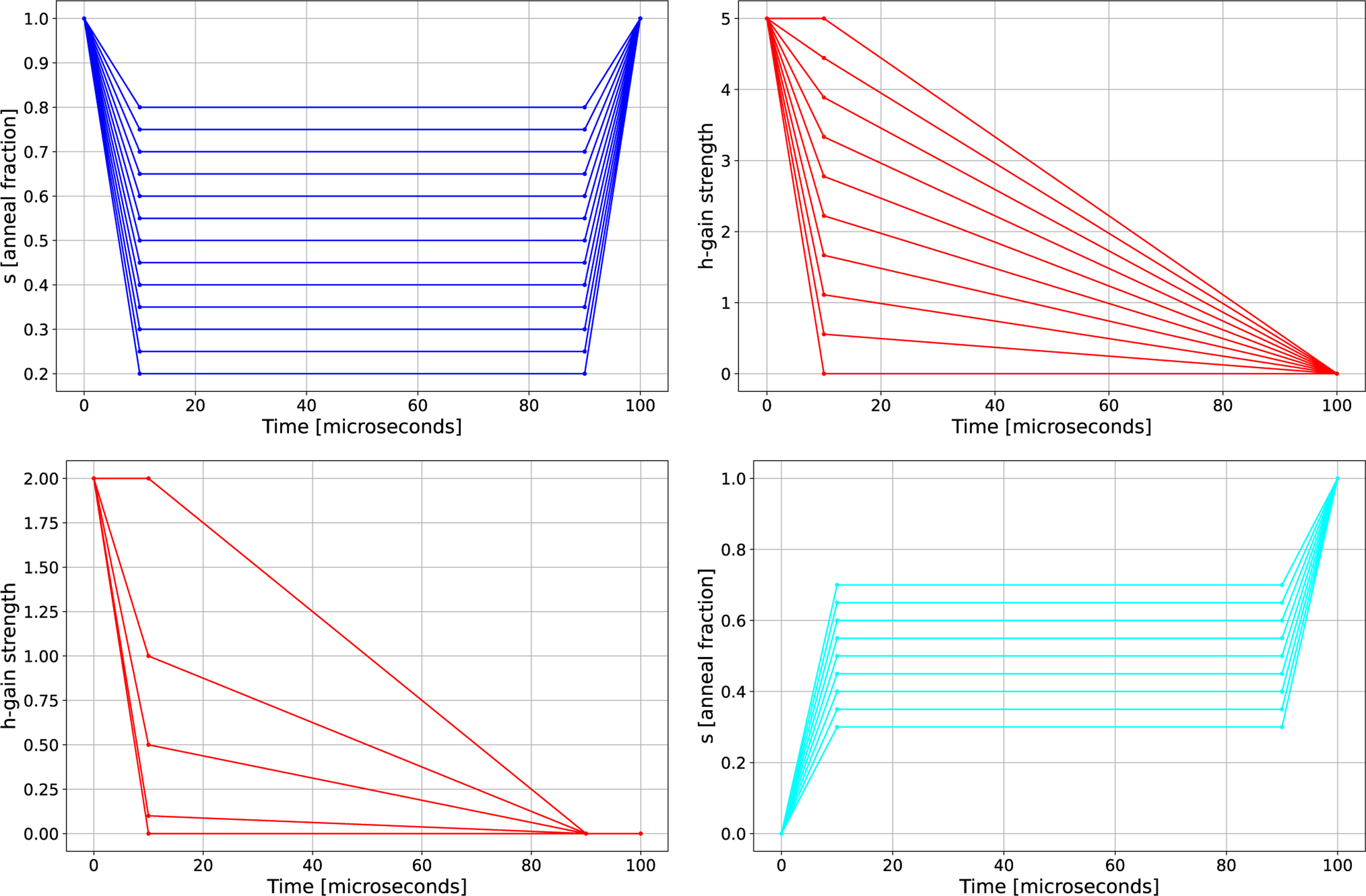
(Top left) Range of RA schedules, (top right) single point varied HG schedule with a fixed annealing time of 50 *μ*s, (bottom left) single point varied HG schedule with an HG strength of 0 at 90 *μ*s, and (bottom right) FA schedules with pauses. The total annealing time for each schedule is 100 *μ*s. The annealing pauses for both RA and FA start at 10 *μ*s.

1. *RA with a single symmetric pause:* Here, we fix the anneal time and time for the ramps, but vary the anneal fraction s at which the pause occurs. Example RA schedules are shown in Fig. 2 (top left subplot).
2. *HG initial state encoding only:* We design the HG schedules to be monotonically decreasing HG value over time, where the first and last points of the schedule are fixed and the middle point can be varied both in terms of the time point and the HG strength. Fig. 2 (top right subplot) shows the functional shape of these HG schedules, where we have fixed the time at which the change occurs to be 10 *μ*s into the 100−μs anneal. The HG schedules are applied at the same time as the default linearly interpolated FA schedule. The earlier in the schedule the HG field is set to 0, the less influence the initial state has on the evolution of the annealing process. Conversely, the later in the annealing process the HG field is set to 0, the greater the influence the initial state has on the evolution of the quantum state. Importantly, the maximum HG field used in Fig. 2 (top right subplot) varies depending on the device, but the plot shows an example where the HG field change occurs at steps linearly spaced between 0 and 5.
3. *Reverse QA schedule, with a symmetric pause, combined with the HG initial state encoding method:* This method involves specifying an RA schedule, as shown in Fig. 2 (top left subplot), and an HG schedule, as shown in Fig. 2 (bottom left subplot). In this case, we modify the HG schedule slightly so that it reaches an HG strength of 0 at the same time that the RA schedule begins to move back toward the readout state (s=1). However, the HG schedule is still monotonically decreasing.
4. *FA schedule with a symmetric pause combined with the HG initial state encoding method:* In this case, we combine an FA schedule with a symmetric pause as shown in Fig. 2 (bottom right subplot) with an HG schedule shown in Fig. 2 (bottom left subplot). As in the previous case that combined FA and HG, the HG schedule here is constructed such that it reaches an HG strength of 0 at the same time in the annealing process that the FA schedule stops pausing before it continues up to s=1.

Each of these four initial state encoding methods is tested on the three D-Wave quantum annealers shown in Table 1. We apply QEMC to random Ising models (see Section I), where the couplers in each problem are designed to match the hardware graph of each the three QPUs in Table 1, meaning that the test Ising models use the entire quantum annealer chip making the search space for the optimal variable assignments quite large. An example of those hardware graphs is given in Appendix B.

We test three different weight precision levels: ten linearly spaced weights between −1 and 1 (not including 0), 100 linearly spaced weights between −1 and 1, and 200 linearly spaced weights between −1 and 1 (excluding 0 so that all coefficients are nonzero). The purpose of this approach is to push the limits of the precision with which the Ising coefficients can be mapped onto the QA hardware of the D-Wave devices. All the problem instances we test only contain quadratic terms, thus leaving the linear terms free to encode the initial state in our proposed HG state encoding method (see Section III-B).

## IV. EXPERIMENTAL ANALYSIS

This section reports on a variety of experiments conducted to assess the performance of both RA and HG, the combined of RA+HG, as well as QEMC for improving a planted solution. After introducing the experimental setting (see Section IV-A), the main experiments are divided into three subsections. First, we investigate two important NP-hard problems: the weighted maximum cut problem (see Section IV-B) and the weighted maximum clique problem (see Section IV-C). Then, the assessment of QEMC in Section IV-D is performed on random spin glass models.

The experiments on minor embedded combinatorial optimization problems in Sections IV-B and IV-C are performed using a D-Wave 2000Q quantum annealer located at the Los Alamos National Laboratory with chip id DW_2000Q_LANL. The experiments in Section IV-D are performed using three other D-Wave quantum annealers with chip ids DW_2000Q_6, Advantage_system4.1, Advantage_system6.1.

### A. EXPERIMENTAL SETTING

The structure of Sections IV-B and IV-C is identical: we first fix the scaling constants in (6) for HG before we determine a suitable annealing duration for applying each of the three methods. Afterward, we employ Bayesian optimization to determine the best anneal schedule, parameterized as described in Section III-E. Once both the annealing duration and the anneal schedule are found for each of the RA, HG, and RA+HG methods, we evaluate all the three techniques with respect to either the cut value (for the edge-weighted maximum cut problem) or the clique weight (for the vertex-weighted maximum clique problem).

The experiments on weighted maximum cut and weighted maximum clique problems used Erdős–Rényi random graphs [48] with probability/density parameter p, where p∈{0.1,0.2,…,0.9}. Once the Ising model coefficients for the maximum cut or maximum clique problem are computed for each test graph, we embed it using the tool minorminer [49], [50] (generating random minor embeddings, produced with all default minorminer parameters) using a chain strength value of 2 and the default settings on the D-Wave devices. Note that, in general, without custom algorithms for structured minor embeddings [51], random minor embeddings are difficult to compute and NP-hard in general [17], [52], [53]. Minor embeddings represent a logical problem graph on the hardware graph by linking together chains of qubits with strong ferromagnetic couplers to form a logical variable state, which ideally retains the same state during the annealing process and at readout.

In order to have a baseline truth for comparing RA, HG, as well as RA+HG, we proceed as follows. We generate random graphs with 65 vertices, which is the largest random all-to-all minor embedding that can fit onto the DW_2000Q_LANL chip. This graph size ensures that even if the QUBO or Ising model is quite dense, there will still be a computable minor embedding. For each density, ten of those graphs are fixed, along with their random minor embeddings; the random minor embeddings are computed individually for each graph, allowing the minorminer tool to reduce minor embedding chain lengths for sparser QUBOs or Ising models, and that embedding is then reused when that graph is sampled again. We then perform 1000 anneals of duration 1 *μ*s for each of the test instances. The best solution among those anneals is then taken as the baseline. When testing RA, HG, and RA+HG, all the values we report are averages over those ten graphs. Moreover, we generate another set of ten graphs for each density to use as a validation set. All samples (for the minor embedded maximum clique and maximum cut problems) are unembedded using the default method in the D-Wave SDK, which applies majority vote to chains that contain qubit state measurements that disagree on the logical state of the variable.

Moreover, we employ the bayes_opt tool of [35] using the following parameters: the number of points for random exploration is set to init_points=100, the number of iterations for optimization is set to n_iter=200, and the noise level is set to alpha=0.01. The parameter alpha indicates to the optimizer how noisy the optimization landscape is. Since D-Wave samples are quite noisy (in part simply due to the finite sampling effect), we observed that setting alpha to a higher value, such as 0.01, is favorable. However, we observe that large values of alpha seem to cause an error in the optimizer, while smaller values lead to insufficient exploration of the optimization landscape.

In the experiments of Section IV-D, we investigate random spin glass models, which fit the D-Wave topology of the three annealers given in Table 1, sampled using iterated RA (also known as QEMC). The annealing time is always 100 *μ*s, and we read out 1000 anneals. The readout thermalization and programming thermalization times were both set to 0 *μ*s. The Boolean option to reduce intersample correlation was enabled. For experiments involving RA, the option reinitialize_state was enabled. All schedules with a pause had a pause duration of 80 *μ*s, with 10 *μ*s for the ramp up and ramp down parts. The initial state for each random spin glass instance was determined by running a single job of 1000 anneals at 100−μs annealing time, using a standard FA schedule. The sample with the best energy was used as the starting point for all iterative procedures. We repeat this across the different D-Wave devices (see Table 1) and the different random spin glass coefficient precisions. In all QEMC applications, we used exactly 20 iterations, where the best sample found at the previous iteration seeds the encoded states for the current iteration.

The maximum HG strengths that can be applied are device dependent. For DW_2000Q_LANL, the maximum HG strength is 5; for DW_2000Q_6, the maximum HG strength is 5; for Advantage_system4.1, it is 3; and for Advantage_system6.1, it is 4. Note that although not used in these experiments, the maximum HG field strengths are sign-symmetric, so, for example, HG schedules with strengths of −5 are also valid for DW_2000Q_LANL and DW_2000Q_6. These maximum HG field strengths affect the range of possible HG schedules that can be applied for the HG state encoding methodology. Typically, for the QEMC experiments, we will hold the HG schedules constant across the tested devices in order to perform a direct comparison.

### B. WEIGHTED MAXIMUM CUT PROBLEM

This section focuses on the edge-weighted maximum cut problem, defined as follows. Given an undirected graph G=(V,E) with edge weights w(e) for each edge e=(u,v) connecting two vertices u, v∈V, we define a *cut* to be any partition of V into the disjoint union $C_{1}\cup C_{2}$, where $C_{1}\subseteq V$ and $C_{2}=V\backslash C_{1}$. The set of cut edges, called *cutset*, is defined as $𝓔=\left\{e=(u,v)\in E:u\in C_{1},v\in C_{2}\right\}$ and its weight is $\sum_{e\in 𝓔}w(e)$. The *weighted maximum cut problem* asks to find a cutset of maximum weight. The Ising formulation of the weighted maximum cut problem is obtained by modifying the (unweighted) formulation in [54], resulting in

$$Q_{cut}(x)=\sum_{(i,j)\in E}w((i,j))\cdot x_{i}x_{j}$$

where $x_{i}$, $x_{j}\in \{-1,+1\}$. Since the Ising formulation of the maximum cut problem does not have linear terms, no slack variable z is needed in the Ising formulation of (6). The scaling constant $\alpha_{1}$ for HG in (6) is given in Appendix A.

For the weighted maximum cut problem graphs, random graphs are generating with edge probability p∈{0.1,0.2,…,0.9} and uniformly drawn edge weights in (−1,1).

Before comparing RA, HG, and RA+HG, a series of tuning parameters given in Section III-E have to be determined. Details on these experiments can be found in Appendix C. The values of the scaling constants $\alpha_{1}$ and $\alpha_{2}$ we employ can be found in Table 2. Moreover, we observe that an annealing duration of 2000 *μ*s works best for RA, while a 1−μs annealis best for HG (see Table 3). When using RA+HG, we likewise employ an annealing time of 2000 *μ*s. The anneal schedules we use are given in Appendix C–B.

#### 1) COMPARISON OF RA, HG, AND RA+HG

**FIGURE 3.**
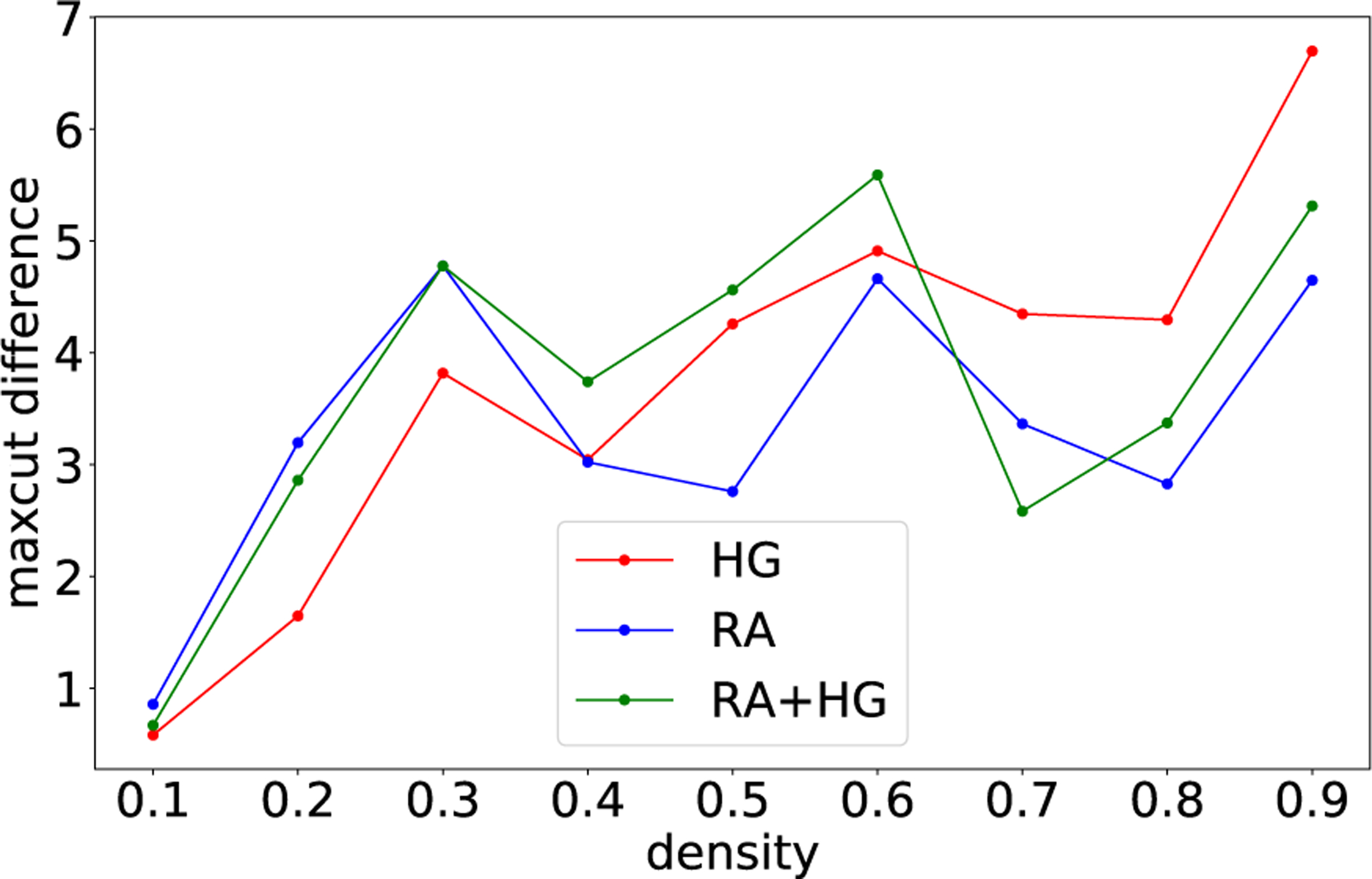
Comparison of RA, HG, and RA+HG with respect to the maximum cut improvement (the difference in maximum cut to the baseline value) per random graph density (x-axis). Best schedules obtained via Bayesian optimization. Plot uses a set of ten new (unseen) test graphs. Computed on DW_2000Q_LANL.

Having determined best schedule parameters for RA, HG, and RA+HG, we run the experiment again on ten new graphs (per density) using these schedules. Fig. 3 shows results of this experiment. We observe that neither technique is uniformly better than the others. RA seems to be best for low densities, while HG and RA+HG perform best for high-density graphs.

#### 2) BEST SCHEDULES FOR RA, HG, AND RA+HG

It is interesting to look at the shape of some of the optimal schedules for RA and HG found by the Bayesian optimization. For this purpose, we visualize one example of a schedule for RA+HG.

**FIGURE 4.**
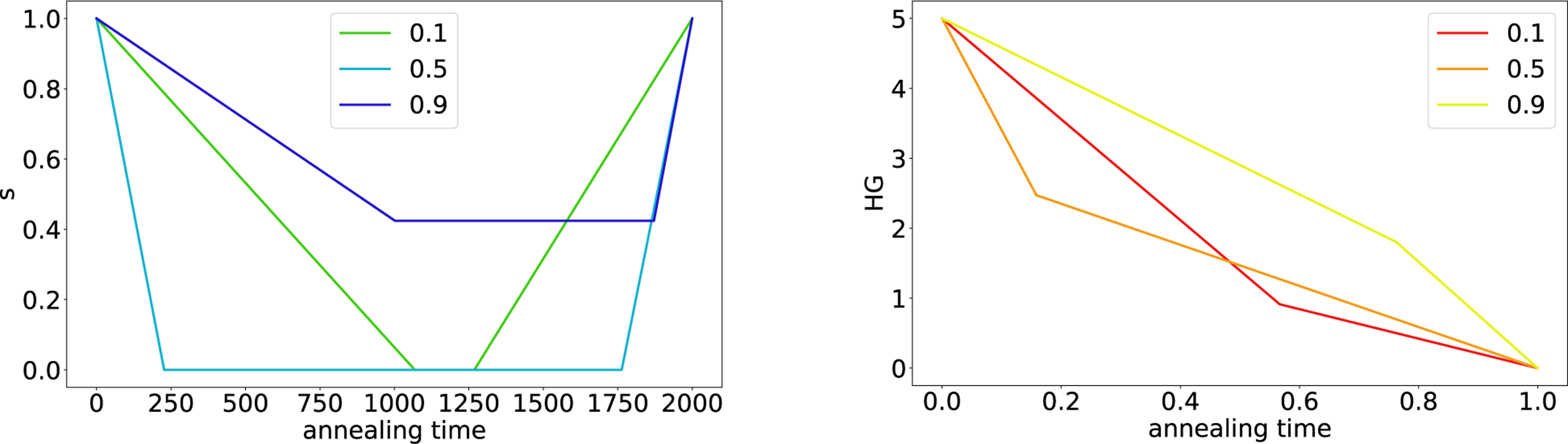
Maximum cut problem. Best schedules for (left) RA and (right) HG for three different densities each, optimized for maximum cut difference. Each line is the best schedule for one density. These schedules were computed using the Bayesian optimization approach, on DW_2000Q_LANL.

Fig. 4 shows the best schedules for RA and HG colorcoded by density. For improved readability, we only display the schedules for p∈{0.1,0.5,0.9}.

We observe a pattern for the RA schedules in Fig. 4 (left). In particular, when optimizing for maximum cut difference, RA schedules for low densities decrease down to an anneal fraction of zero, followed by a pause until roughly the midpoint of the anneal. In contrast, RA schedules for high densities only decrease to roughly an anneal fraction of 0.5 at the midpoint of the anneal, followed by a pause until almost the full annealing time.

Similarly, a pattern can be observed for the HG schedules in Fig. 4 (right). The HG schedules for low densities seem to have a steeper slope at the start of the anneal and flatten off afterward. In contrast, schedules for high densities seem to be closer to a straight line between the start point (0,5) and the end point (1,0).

An RA+HG anneal can be executed by sending to the D-Wave back end (in this case DW_2000Q_LANL) one RA schedule and one (independent) HG schedule. While the RA aspect is easy to comprehend, the HG one is more difficult to grasp by just looking at the two component schedules because of the way the RA portion affects HG. Specifically, if s=RA(t) and g=HG(t) are the functions determined by the RA and HG schedules, respectively, then the real gain applied at time t to the linear biases in (3) is (B(s)/2)HG(t)=B(RA(t))HG(t)/2, where B(s) is the function from (2).

**FIGURE 5.**
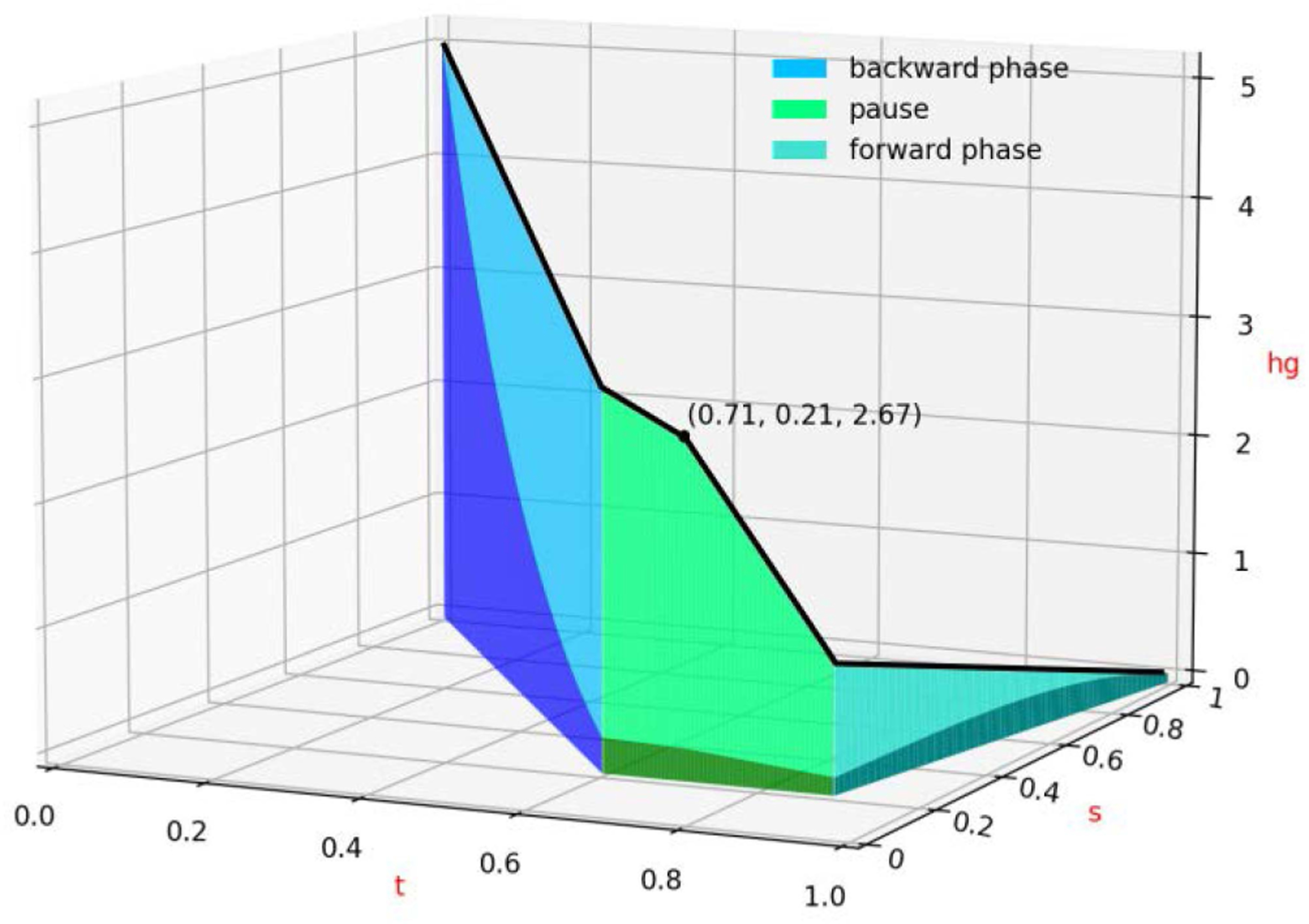
Illustration of an RA+HG schedule. Best schedule found for the weighted maximum cut problems for p=0.3 graph density using Bayesian optimization, run on DW_2000Q_LANL.

Fig. 5 is given to help visualize the effect of an RA+HG schedule and the interplay between the parameters. The time t is normalized in [0,1]. The RA component of the schedule, which has a pause for t∈[0.6,0.89] at s=0.21, can be seen as a projection in the t−s plane. The black line shows the HG values, specifically, the points (t, s(t), hg(t)) for t∈(0,1). The HG schedule, which has middle point at (t,hg)=(0.71,2.67), can also be seen as the lighter color projection in the t−hg plane. The blue, green, and teal colors indicate the backward annealing, pause, and FA phases, respectively. Finally, the cumulative gain applied to the linear biases at each time, which depends on both the values of HG and the annealing coefficient B(s) from (3), is represented by the darker colored portion of the plot. The annotated point shows the value of the HG (2.67) at the middle point of the HG schedule (at 0.71). To simplify the plot, function B(s) has been normalized to [0,1]. We can see that the real gain applied during the pause and forward phases of the RA schedule stays mostly unchanged. These observations are, in a strict sense, valid for experimental setting of Section IV-A only, although we anticipate them to hold true in greater generality for a broader class of problems.

### C. WEIGHTED MAXIMUM CLIQUE PROBLEM

We carry out a similar analysis for the vertex weighted maximum clique problem, defined as follows. For any graph G=(V,E), a clique C is a fully connected subset of vertices, i.e., C⊆V such that C×C⊆E. A maximum clique is a clique in G of maximum size. For the (vertex-)weighted version of the problem, we define a weight w(v) for each vertex v∈V. The weight of a clique is accordingly defined as $w(C)=\sum_{v\in C}w(v)$. The weight of a clique is accordingly defined as The weighted maximum clique problem asks for the clique C⊆V having the largest weight w(C). The QUBO formulation of the weighted maximum clique problem is obtained by modifying the (unweighted) formulation in [55], resulting in

$$-\sum_{n}^{i=1}w(i)\cdot x_{i}+2\sum_{(i,j)\notin E}\max\{w(i),w(j)\}\cdot x_{i}x_{j}$$

where $x_{i}$, $x_{j}\in \{0,1\}$. We can convert the above QUBO formulation into an Ising problem using the equivalence given in [55]. In contrast to the maximum cut problem investigated in Section IV-B, the maximum clique formulation as an Ising model of the form of (1) does contain linear terms. We, thus, introduce a slack variable z to homogenize the linear terms as in (5) and add a new linear term encoding the initial solution as done in (6).

In the following experiments, we choose the vertex weights to be positive and randomly drawn from a uniform distribution in (0.001,1).

As done for the weighted maximum cut problem, a set of parameters has to be determined for RA, HG, as well as RA+HG. Details on these experiments can be found in Appendix D. The values of the scaling constants $\alpha_{1}$ and $\alpha_{2}$ we employ can be found in Table 2. Based on the parameter tuning conducted in Appendix D, we run RA with an annealing time of 2000 *μ*s in the rest of this section, and HG with an annealing time of 1 *μ*s. For RA+HG, we fix the annealing duration at 2000 *μ*s (see Table 4). The schedules can be found in Appendix D–B.

#### 1) COMPARISON OF RA, HG, AND RA+HG

**FIGURE 6.**
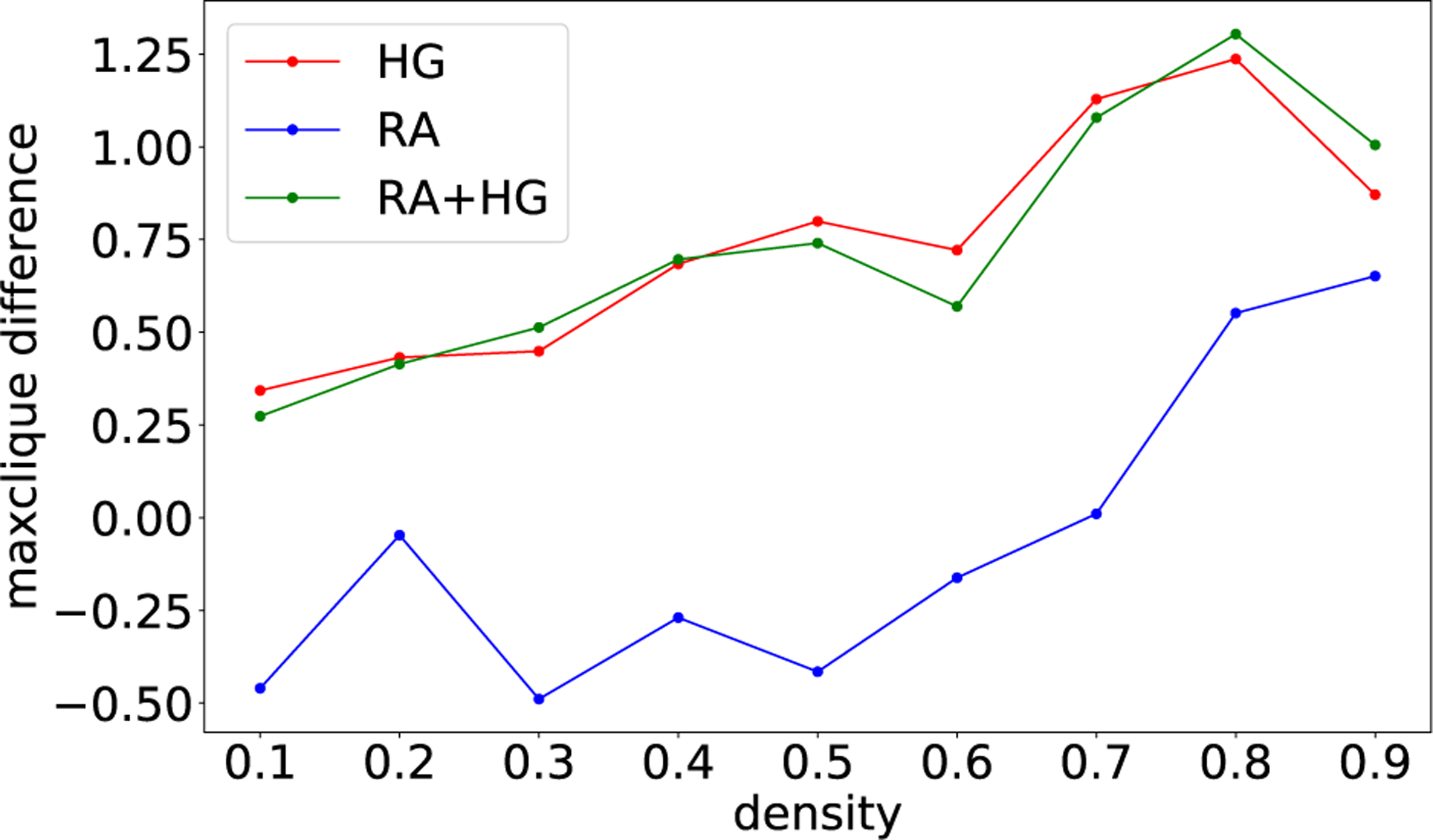
Comparison of RA, HG, and RA+HG with respect to the improvement in maximum clique weight (the difference in maximum clique weight to the baseline value) per random graph density (x-axis). Plot uses a set of ten new (unseen) test graphs. Computed on DW_2000Q_LANL.

As in Section IV-B1,we evaluateRA,HG,aswellasRA+HG after tuning the scaling factors, annealing durations, and schedules. Results are shown in Fig. 6 for ten new problems not used in the training set. We observe that the behavior of all three techniques is consistent: on the new problems, RA performs worst with the exception of graph density corresponding to p=0.9. Both HG and RA+HG perform very similarly and consistently better than RA, although they draw equal with RA for p=0.9.

This behavior is different from the equivalent experiment for maximum cut in Fig. 3, where both HG and RA+HG were only marginally better than RA.

### D. EVALUATION OF QEMC

We finally evaluate QEMC, that is, the technique of iteratively improving the sampled solutions. As outlined in Section III-F, four options are available for planting the solution in iteration. Those are the options evaluated in Sections IV-B and IV-C, namely RA only, an HG field only (using the qubit coefficients to encode an initial state for the anneal), RA combined with HG (where the same initial states is encoded using both linear terms and the RA initial state), and forward annealing with a pause combined with HG initial state encoding. Each QEMC iteration uses 1000 anneals and an annealing time of 100 *μ*s, and we plot the lowest energy state found at each iteration. The lowest energy sample found at each iteration then seeds the encoded state of the anneal for the next iteration (whether it is encoded using HG state encoding or RA). For good parameter choices, we would observe that there is a monotonic decrease in the energy, e.g., the solutions are getting progressively better for the minimization combinatorial optimization problem. Each QEMC experiment (e.g., with each unique Ising model on each device) is initialized with the best solution found from a normal FA (e.g., with a linearly interpolated anneal schedule) from 1000 anneals with an annealing time of 100 *μ*s. Therefore, each set of QEMC experiments begins in exactly the same initial state.

These experiments are conducted on random spin glass models. As remarked in the literature [56], [57], [58], random spin glasses with the Chimera structure have been shown to exhibit a zero-temperature phase transition. Random spin glasses with Chimera connectivity are, therefore, not expected to be computationally difficult to sample from classically, which could be a reason why an advantage of quantum over classical optimization algorithms has not been demonstrated yet for this class of Ising models. However, there is also evidence that general spin glasses on these hardware graphs (Chimera and Pegasus) may provide advantageous sampling for very large system sizes [59].

#### 1) QEMC WITH RA

**FIGURE 7.**
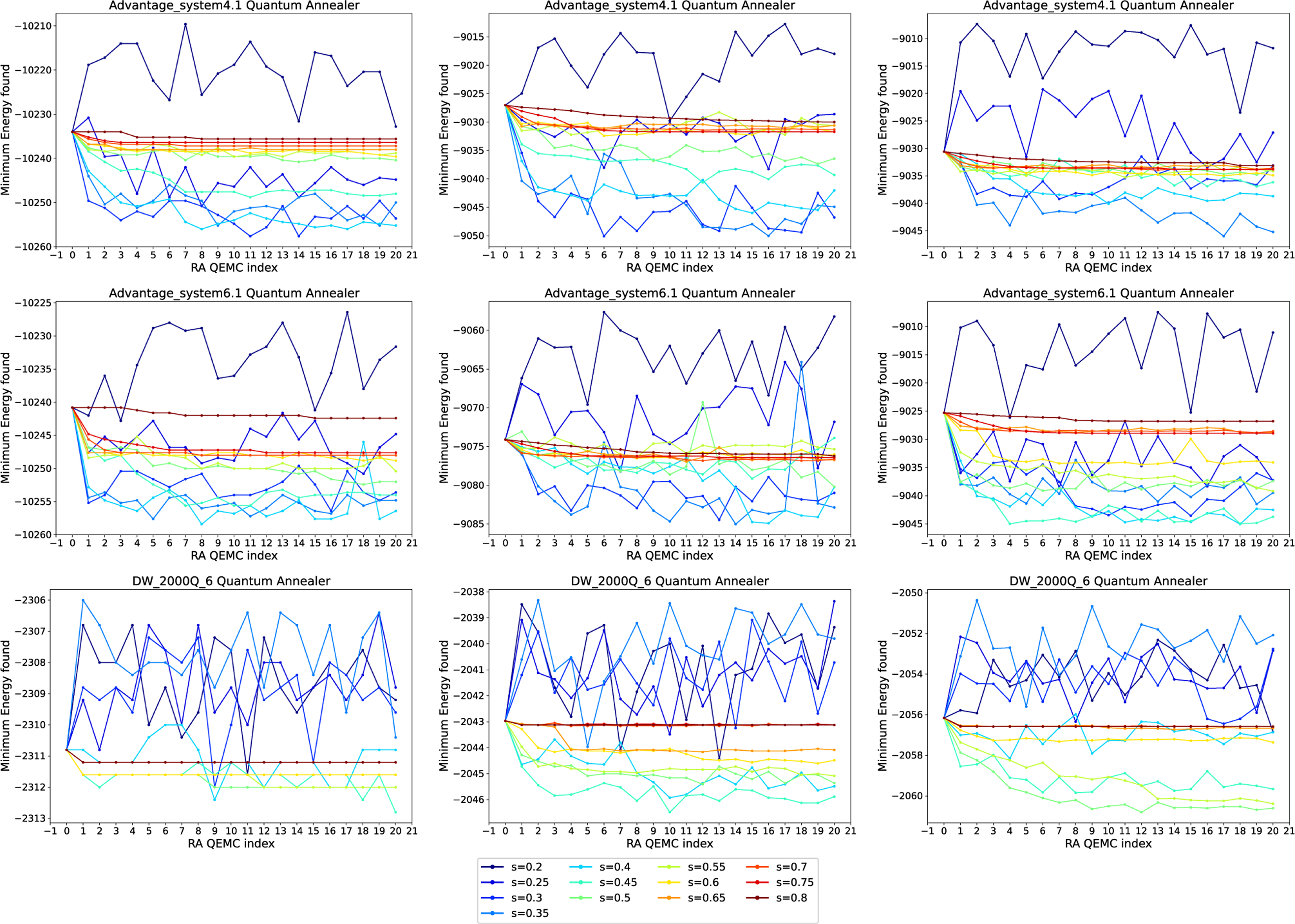
QEMC with RA only, where each subsequent step uses the lowest energy solution found from the previous step. The problem instances are random spin glasses on (top row) Advantage_system4.1, (middle row) Advantage_system6.1, and (bottom row) DW_2000Q_6. Spin glasses generated with linearly spaced precision of (left column) 10, (middle column) 100, and (right column) 200. The curves show different anneal fractions s at which the symmetric reverse anneal is paused at, given in the legend. Annealing time of 100 *μ*s and 1000 anneals per step are used for these experiments.

We first consider QEMC with RA only, meaning that we iteratively refine a solution by encoding the previously found best result using RA. In this experiment, the anneal fraction s at which the pause occurs is varied; we utilize values s∈{0.2,0.25,0.3,…,0.8}. Fig. 7 shows our results for the three quantum annealers given in Table 1 (rows) and spin glass generation mechanisms (columns). Each curve is a run with a different anneal fractions s for RA, given in the legend. The figure displays the minimum energy found in the 1000 anneals in each iteration and for each parameter.

We observe that the behavior of QEMC with RA on Advantage_system4.1 is quite similar to the one on Advantage_system6.1, while results on DW_2000Q_6 differ. For the first two devices, lower anneal fractions are suboptimal, while anneal fractions around s∈[0.3,0.4] perform best. For anneal fractions that are too high, we observe little improvement of the solution over the iterations of QEMC. For DW_2000Q_6, results display a higher degree of volatility, and the values of s yielding the best converge behavior are slightly higher than for the newer annealer generations, which is around s∈[0.5,0.6].

#### 2) QEMC WITH HG STATE ENCODING

**FIGURE 8.**
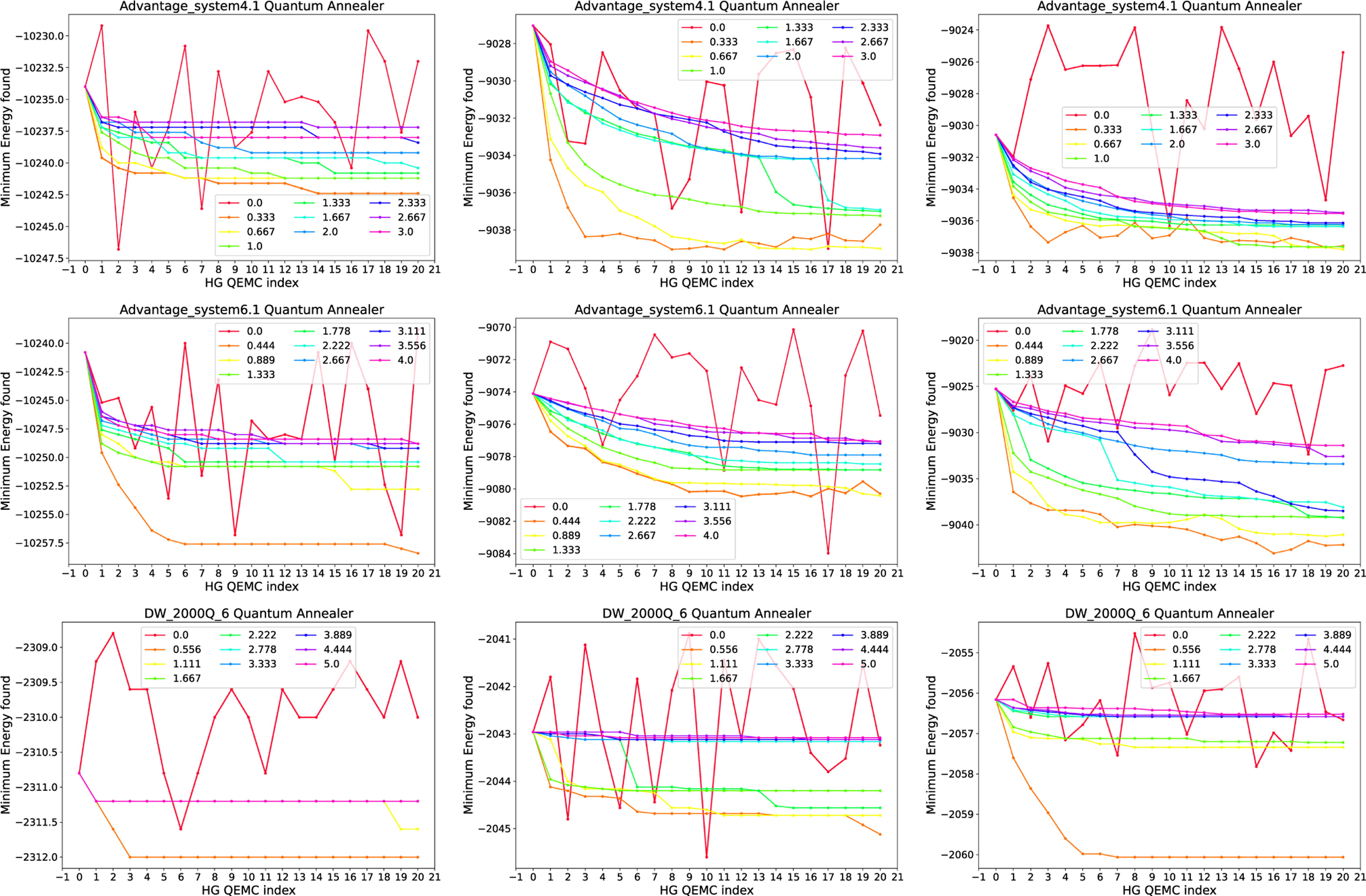
QEMC with HG initial state encoding. Random spin glasses on (top row) Advantage_system4.1, (middle row) Advantage_system6.1, and (bottom row) DW_2000Q_6. Spin glasses generated with linearly spaced precision of (left column) 10, (middle column) 100, and (right column) 200. The curves show different HG strengths h, given in the legend. The single point that is varied in the HG schedules used for these experiments is set to 10 *μ*s into the 100−μs anneal. The maximum HG strength used was set to the maximum allowed on each D-Wave quantum annealer, which is 5 for DW_2000Q_6, 4 for Advantage_system6.1, and 3 for Advantage_system4.2.

Next, we repeat the same experiment in Fig. 8, though now we use HG state encoding, comprising of the HG schedule and programming linear terms on the qubits corresponding to the encoded state, to plant the best previous solution sampled in each iteration. As before, the figure displays the minimum energy found in the 1000 anneals in each iteration and for each parameter. The parameter h is varied in the interval [0,3] for Advantage_system4.1, in [0,4] for Advantage_system6.1, and [0,5] for DW_2000Q_6. The HG strength is set to 0 at 10 *μ*s into the 100−μs anneal, which means that the initial state is strongly encoded during the transverse field dominated portion of the anneal and then is switched off for the rest of the anneal (see the top right subfigure of Fig. 2).

The results in Fig. 8 demonstrate that the HG state encoding technique can be used to execute QEMC. We again observe that the newer generations of the D-Wave annealer, that is, Advantage_system4.1 and Advantage_system6.1, seem to be better suited for QEMC as they show a more stable convergence behavior. For DW_2000Q_6, convergence seems more unstable.

#### 3) QEMC WITH RA AND HG

**FIGURE 9.**
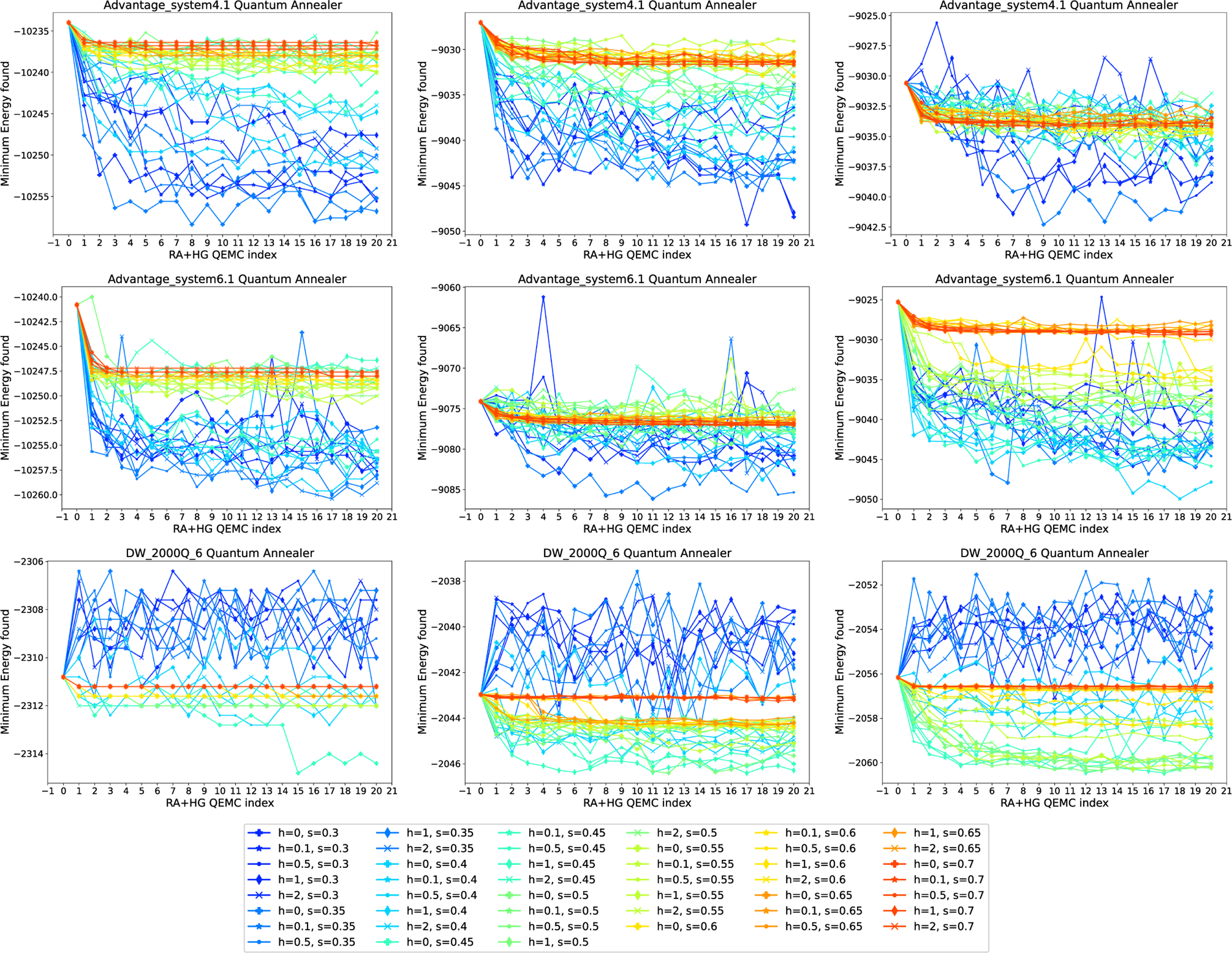
QEMC using RA and HG combined. Random spin glasses on (top row) Advantage_system4.1, (middle row) Advantage_system6.1, and (bottom row) DW_2000Q_6. Spin glasses generated with linearly spaced precision of (left column) 10, (middle column) 100, and (right column) 200. The curves show different combinations of both the symmetric pause anneal fractions s for RA and the HG strength h, given in the legend. Note that h in the legend corresponds to the HG strength specified for at 10 *μ*s into the 100−μs anneal, but the initial HG field is consistently set to be initialized an HG strength of 2 (which in particular means these schedules are compatible with all three quantum annealers in Table 1).

As before, we can also combine RA and HG and use it to iteratively plant solutions before each anneal. This is investigated in Fig. 9, which displays the minimum energy found in the 1000 anneals in each iteration and for each parameter combination of the anneal fraction s for RA and HG strength h. Here, s is varied in the interval [0.3,0.7], and h is varied in [0,2] (which ensures that these schedules are compatible with all three of the tested quantum annealers).

The results in Fig. 9 are consistent with the previous ones reported in Sections IV-D1 and IV-D2. The newer annealer generations seem more suited for an application of QEMC, and across both Advantage_system4.1 and Advantage_system6.1, we observe a consistent improvement of the initial solution when applying QEMC. Interestingly, the best parameter combinations seem to depend more on the choice of s than the choice of h, as roughly s∈[0.35,0.5] in connection with any h yields best results. On DW_2000Q_6, the application of QEMC works less well, though an improved behavior can be seen for higher precision of the mapped Ising coefficients (right-hand column of plots).

#### 4) QEMC WITH FORWARD ANNEALING WITH A PAUSE AND AN HG FIELD

The schedule of forward annealing can be modified to introduce a pause during the anneal. As shown in earlier works [60], [61], [62], using such a pause in the anneal schedule has the potential to considerably increase the probability of successfully finding the ground state of the problem Hamiltonian.

In this case, we can use the HG initial state encoding method to specify an initial state, but then also use a forward annealing schedule with a pause, as opposed to the standard linearly interpolated schedule. The reasoning is that in other contexts, it has been shown that pausing can improve forward and reverse QA. Here, we can utilize the HG field to reinforce that initial state, while the pause is occurring in order to guide the iterative sampling toward monotonic solution improvement. The unknown thing a priori is what HG field strength, and pause parameters, will work well for this—and we also do not know how this might compare against the combined RA and HG initial state encoding method. This method is the same as that shown in Section IV-D2, but the anneal schedule is modified instead of using the standard linearly interpolated anneal schedule.

**FIGURE 10.**
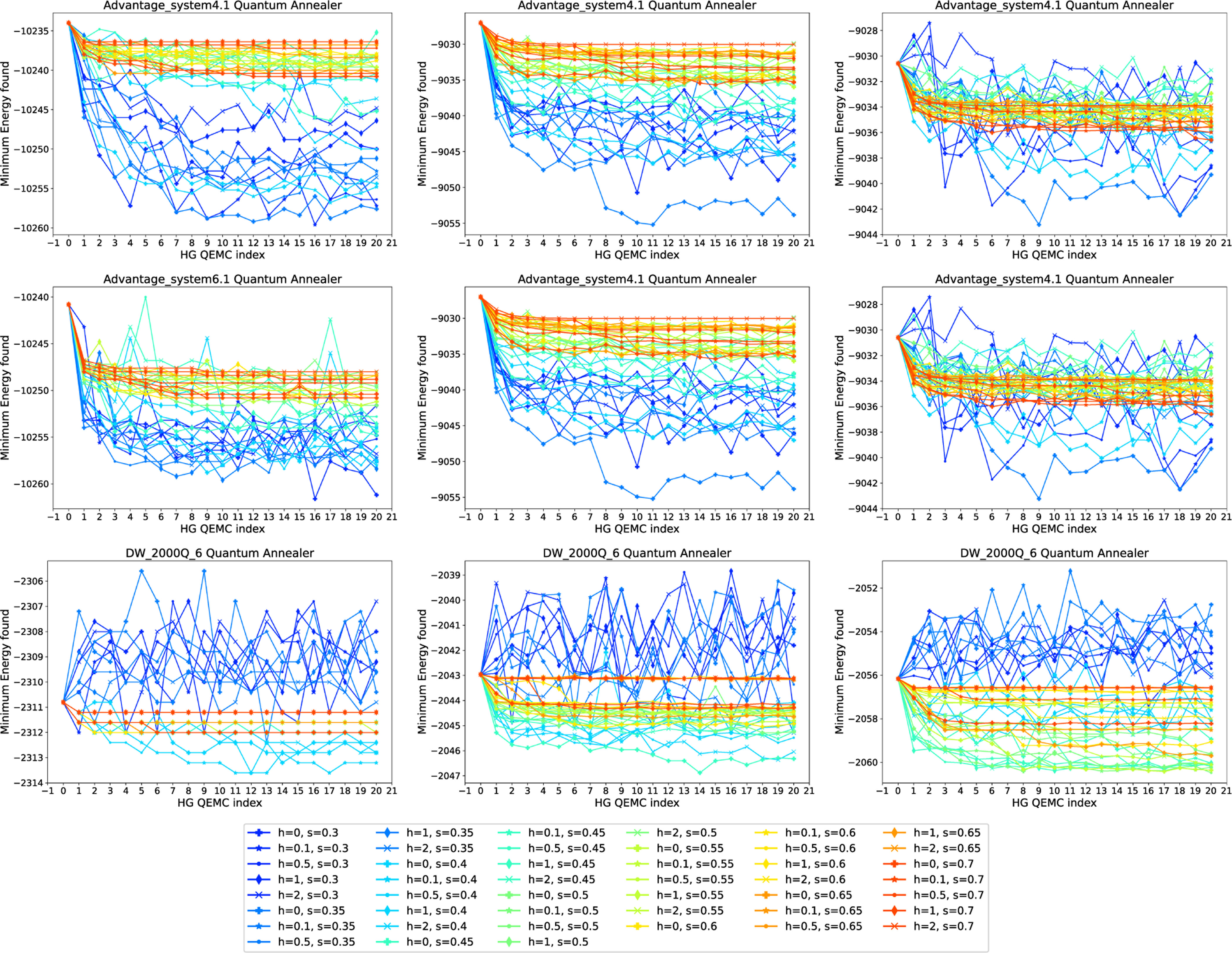
HG initial state encoding, with an FA symmetric pause at varying anneal fractions. At each iteration, and parameter combination, the minimum energy found in the 1000 anneals is plotted as a function of iteration. The HG strength at which the schedule begins to monotonically decrease (bottom left subplot of Fig. 2) is varied, along with the anneal fraction at which the symmetric pause occurs (bottom right subplot of Fig. 2) in the FA schedule. Random spin glass on (top row) Advantage_system4.1, (middle row) Advantage_system6.1, and (bottom row) DW_2000Q_6. Left column random spin glass has linearly spaced precision of 10. The middle column has linearly spaced precision of 100, and the right column has linearly spaced precision of 200. The FA pause fraction is varied, along with the HG strength; the legend shows which of these parameters are varied for each of the different lines in the plots. Note that h in the legend corresponds to the HG strength specified for at 10 *μ*s into the 100−μs anneal, but the initial HG field is consistently set to be initialized an HG strength of 2 (which, in particular, means that this schedule can applied to all three quantum annealers).

The results for using this variant of iterative solution encoding is shown in Fig. 10. As before, s is varied in the interval [0.3,0.7], and h is varied in [0,2]. Notably, the optimal schedule combination of HG schedules and forward annealing schedules (with pauses) is not consistent across the different quantum annealers or Ising models. For Advantage_system4.1 sampling the precision 10, 100, and 200 Ising models, the best parameter choices include the pause anneal fraction being in the range of 0.3–0.4, and the h strength being set to 0–10 s. This shows that the initially very strong HG field that is applied in the first 10 *μ*s of the anneal while the transverse field terms are still dominating the system is enough to seed the anneal and guide toward lower energy states, and provides a reasonable iterative energy improvement when used with QEMC. A similar result is seen for Advantage_system6.1. For DW_2000Q_6 sampling the precision 10 Ising models, s=0.4 and h=0.5−2 give reasonable improvements in energy as a function of iteration. For DW_2000Q_6 sampling the precision 100 and 200 Ising models, the parameters of s=0.45 and h=0−2 are best.

## V. DISCUSSION

In this contribution, we investigated two techniques suitable to encode an initial solution prior to the anneal on the D-Wave 2000Q quantum annealer. The two techniques are the RA feature of the D-Wave device, as well as our own method based on the HG feature.

Since the two techniques rely on a variety of tuning parameters, we conduct extensive testing to determine suitable annealing times, parameters, and schedules. Using optimized sets of parameters, we compare both methods on both the maximum cut problem (whose Ising formulation does not have linear terms, thus making our HG technique directly applicable), as well as the maximum clique problem (for which we have to transform the Ising model first). Afterward, we explore the suitability of both methods in a straightforward technique, iterated QA also referred to as QEMC. We summarize our findings as follows.

1. The HG feature of D-Wave quantum annealers can be used to implement the QEMC algorithm. We demonstrate this using hardware-native spin glass instances. This type of iterative sampling on whole-chip quantum annealers has not been done before, but both the RA and HG version of this could be applied to QA chip native Ising benchmarking such as what is demonstrated in [63].
2. The best annealing durations for RA and HG seem to be very problem dependent. However, there is a consistent pattern in the anneal schedules for the weighted maximum clique and weighted maximum cut. Specifically, for graphs of lower density, RA schedules with an early and longer pause at a low anneal fraction are advantageous, whereas for higher densities, a shorter pause at an anneal fraction of around 0.5 seems better. For HG, the optimal schedules are close to the line connecting (0,5) and (1,0) independently of the density. There are important differences between the maximum cut and maximum clique problem formulations, specifically that the maximum clique QUBO formulation uses the complement of the problem graph edgeset, which means that the maximum clique QUBOs are sparser for denser graphs. This is not the case for maximum cut, and therefore, this structural different could contribute to differences observed when sampling the two problems.
3. The scaling constants can be found successfully via Bayesian optimization.
4. In the minor embedded weighted maximum clique experiments, the quantum annealer almost always returned samples with value for the slack variable z=1 for the optimized HG schedules. Having z=1 is necessary for our technique to work, but we did not expect it to happen so often. One possible explanation is that the HG bias helps guiding the anneals toward solutions with z=1. We also observe that z=1 occurs with much lower frequency for nonoptimal HG schedules.
5. We conclude that our technique to plant initial solutions with the help of the HG feature, as well as RA+HG, seems to be a viable alternative to RA.

This article leaves considerable scope for future work.

1. Determining where possible how close the quantum annealer comes to finding the global optimal solution for whole-lattice spin glass instances (or even just known-best solutions), for example, what is studied in [63], in particular with the addition of iterative sampling using RA or HG state encoding.
2. In this work, we only considered RA schedules with two points defining a pause, and HG schedules with one or two points. However, more complicated schedules for both RA and HG are possible, including other annealing times and RA+HG schedules with more points.
3. The HG initial state encoding technique we have proposed can be applied to many more interesting problems, such as graph partitioning, the traveling salesman problem, minimum vertex cover, or graph coloring. In addition, many of those problems themselves exist in different variants, including unweighted, vertex-, or edge-weighted formulations.
4. We used the Bayesian optimization framework of [35] in a rather ad hoc way. Tuning the parameters of the Bayesian optimization, in particular with the aim to make the optimization more robust against the noise in the D-Wave samples, could further improve the optimized parameters and schedules we report.
5. When applying the HG state encoding method to the minor embedded weighted maximum clique cases, in the cases where z does not always equal 1, one could observe if the proportion of anneals where z=1 is higher for RA+HG in comparison to HG only, assuming that all other variables are held constant. This is conjectured to be true because in RA+HG, the value of z is reinforced by the initial state of RA.
6. Both the HG schedule and the anneal schedule can have many more points specified, allowing for very complex anneal schedules. Generally, complex anneal schedules have not been investigated in detail, for example, by repeatedly turning off and on a specific state specified by the linear terms.
7. The quantum alternating operator ansatz [64], [65] algorithm, which is a hybrid classical–quantum algorithm that operates on universal gate model quantum computers, can be warm-started [46]. Therefore, it can be used to iteratively improve solution quality in a similar fashion to QEMC.

## Figures and Tables

**TABLE 1.** Summary of D-Wave QEMC, or Iterated RA, Experiments

D-Wave device chip ID	Topology name	Available qubits	Available couplers
DW_2000Q_6	Chimera $C_{16}$	2041	5974
Advantage_system4.1	Pegasus $P_{16}$	5627	40279
Advantage_system6.1	Pegasus $P_{16}$	5616	40135

Note that each of these three devices has some hardware defects on the qubit topology, which causes the available hardware (qubits and couplers) to be smaller than the ideal graph lattice structure.

**TABLE 2.** Best Scaling Factor(s) for HG State Encoding as a Function of the Random Graph Density, for the Minor Embedded Weighted Maximum Clique and Maximum Cut Problems

Density	Maximum cut	Maximum clique	
	$\alpha_{1}$	$\alpha_{1}$	$\alpha_{2}$
0.1	0.16	0.3271	0.2997
0.2	0.23	0.2709	0.8897
0.3	0.51	0.1997	0.5401
0.4	0.48	0.1542	0.9246
0.5	0.41	0.3467	0.2473
0.6	0.48	0.2602	0.7586
0.7	0.63	0.0292	0.7455
0.8	0.93	0.0334	0.0210
0.9	0.33	0.0370	0.5121

Left: Parameter $\alpha_{1}$ of the maximum cut problem. Right: Parameters $\alpha_{1}$ and $\alpha_{2}$ of the maximum clique problem.Data from DW 2000QLANL.

**FIGURE 11.**
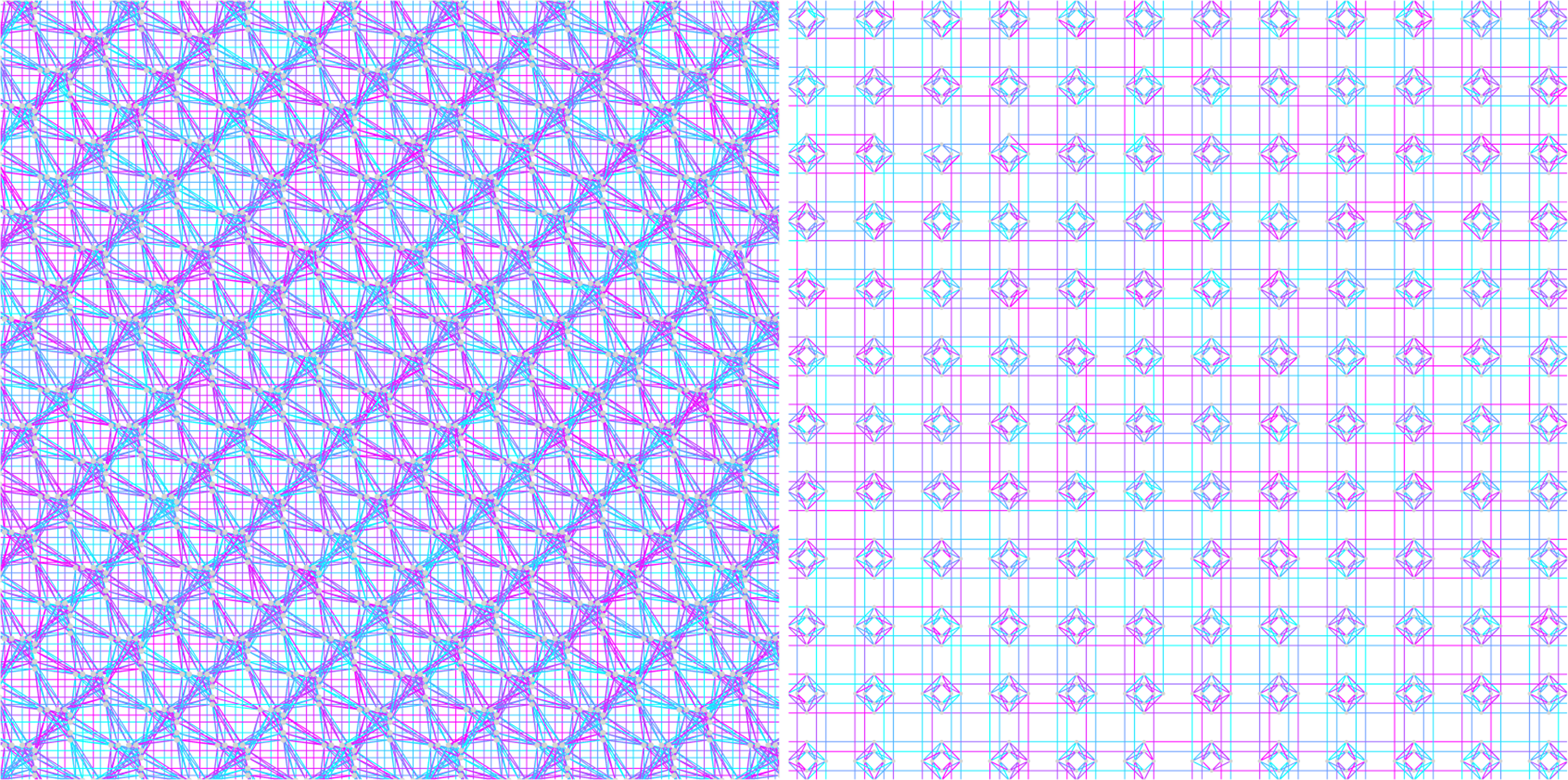
Cropped hardware graphs (edges of the chip are not shown) of the hardware connectivity for embedded spin glass instances on (left) Advantage_system4.1, both of which are Pegasus $P_{16}$ graphs with hardware defects, and (right) DW_2000Q_6 which is a Chimera $C_{16}$ graph with hardware defects. The values of the quadratic coefficients are color coded from (light blue) −1 to (purple) +1.

**FIGURE 12.**
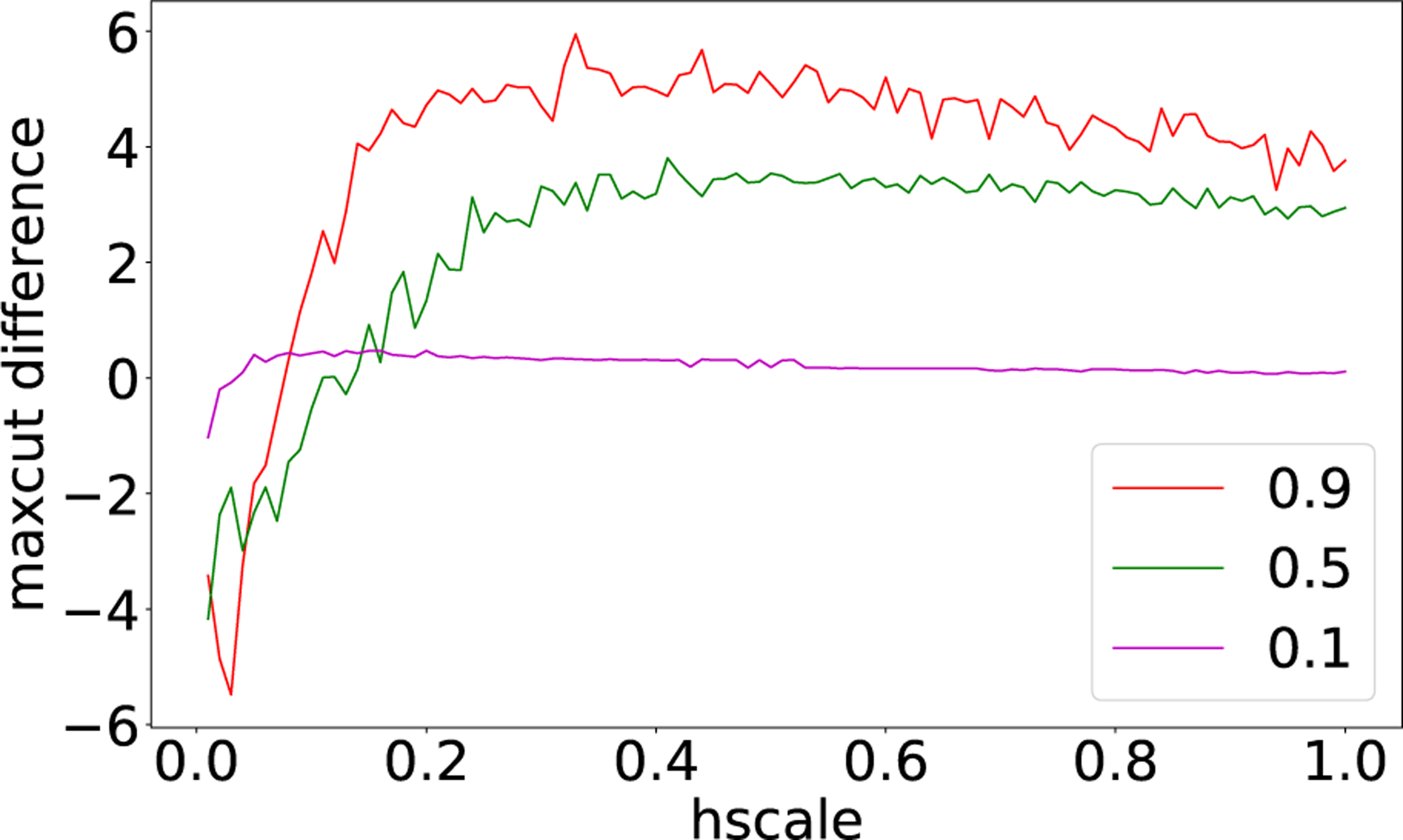
Maximum cut problem. Difference in the maximum cut value to the baseline as a function of the HG scaling factor $\alpha_{1}$. The goal is for the value on the y-axis to be maximized, meaning that the tuned scaling factor improves the weight of the cut found. Data from DW_2000Q_LANL.

**FIGURE 13.**
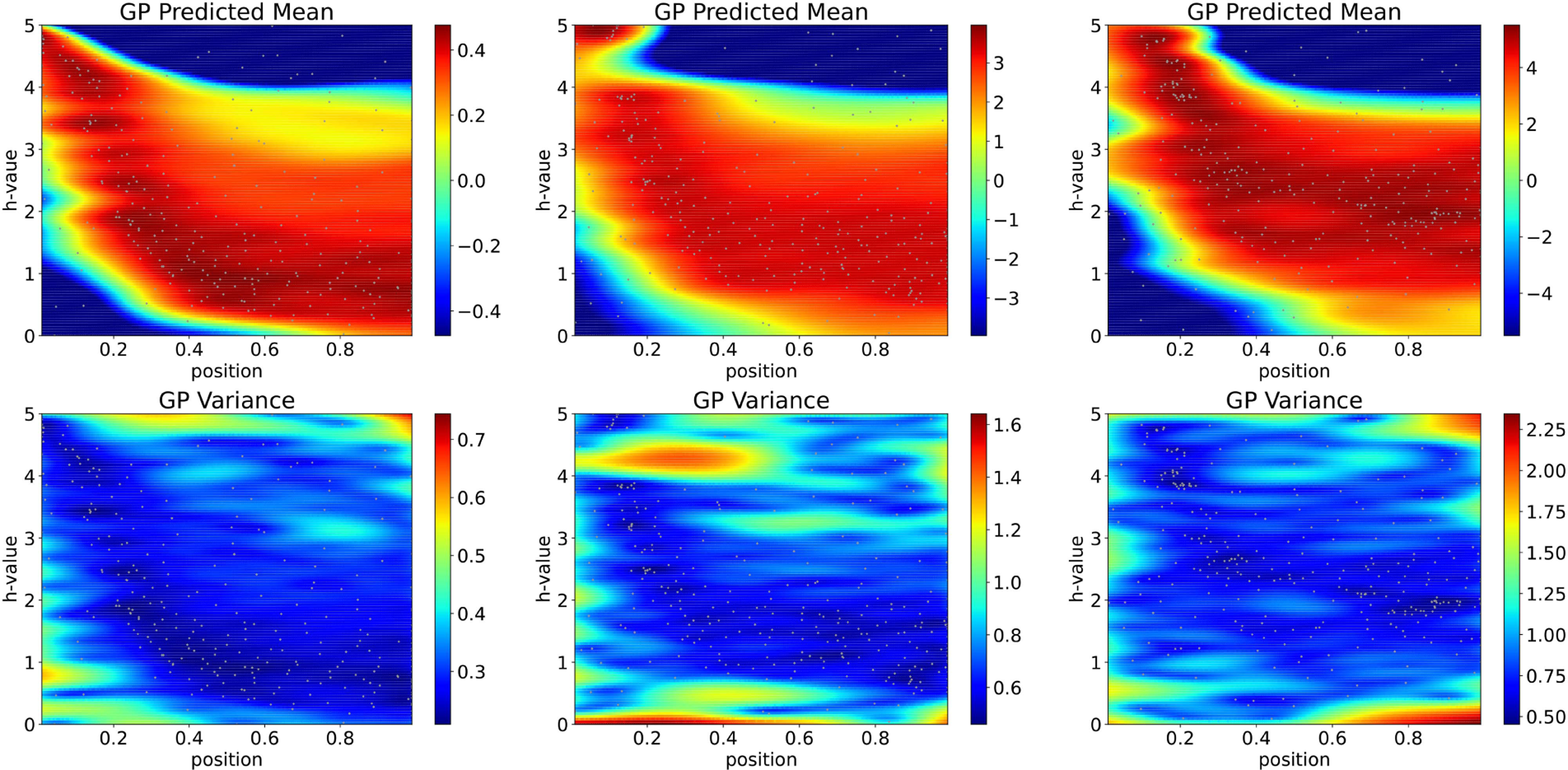
Bayesian optimization of the HG schedule for the weighted maximum cut problem on random graphs of density (left column) 0.1, (middle column) 0.5, and (right column) 0.9. (Top subplots) Mean and (bottom subplots) variance of the Gaussian process (GP) used by the Bayesian optimizer, where the goal is to maximize the objective function (e.g., red regions mean that the constructed HG schedule resulted in better cut values being sampled). The parameters of the HG schedule for maximum cut are visualized as a heatmap for an annealing time of 1 *μ*s. Top shows the maximum cut size improvement as a function of g(t)∈[0,5] [see (3)] on the y-axis, where t is the position in the schedule (x-axis). The small gray dots indicate the points where the objective function was evaluated. Each heatmap is computed from a total of 300 function calls (100 random points and 200 optimization steps). Data from DW_2000Q_LANL.

**FIGURE 14.**
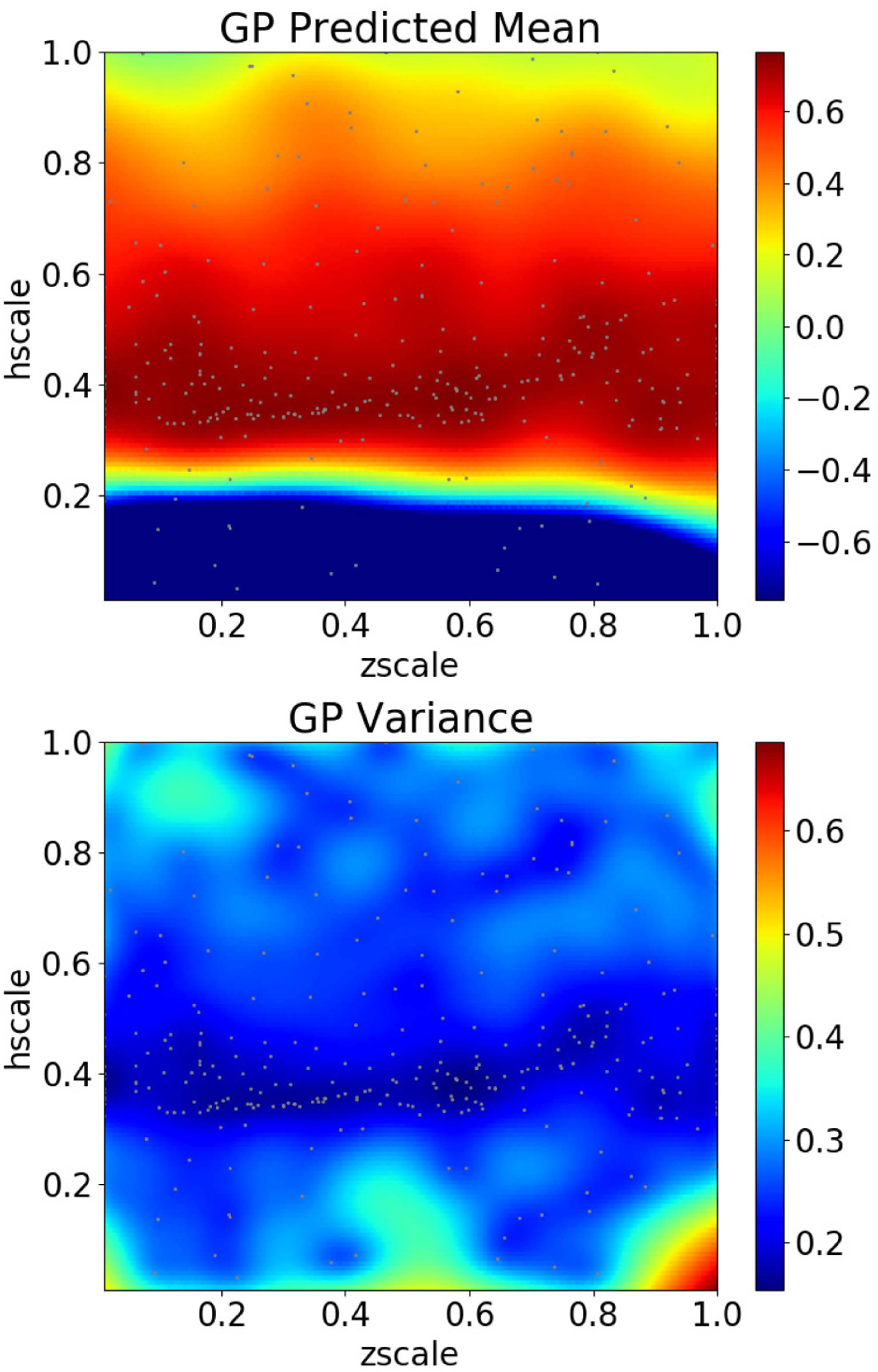
(Top) Mean and (bottom) variance of the Gaussian process (GP) used by the Bayesian optimizer. Landscape encoded in $\alpha_{1}$ (h-scale on the y-axis) and $\alpha_{2}$ (z-scale on the x-axis) for a graph density of p=0.5. The small gray dots indicate the points where the objective function was evaluated. Data from DW_2000Q_LANL.

**FIGURE 15.**
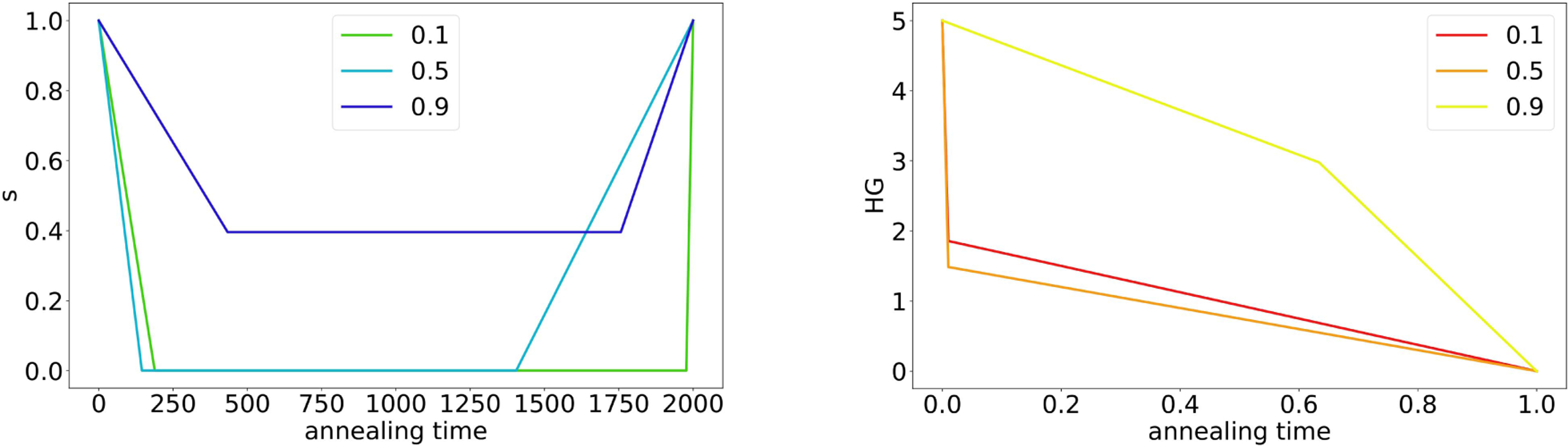
Weighted maximum clique problem. Best schedules for (left) RA and (right) HG optimized for maximum clique weight. Each line is the best schedule for one density. These schedules were computed using the Bayesian optimization approach on DW_2000Q_LANL.

**FIGURE 16.**
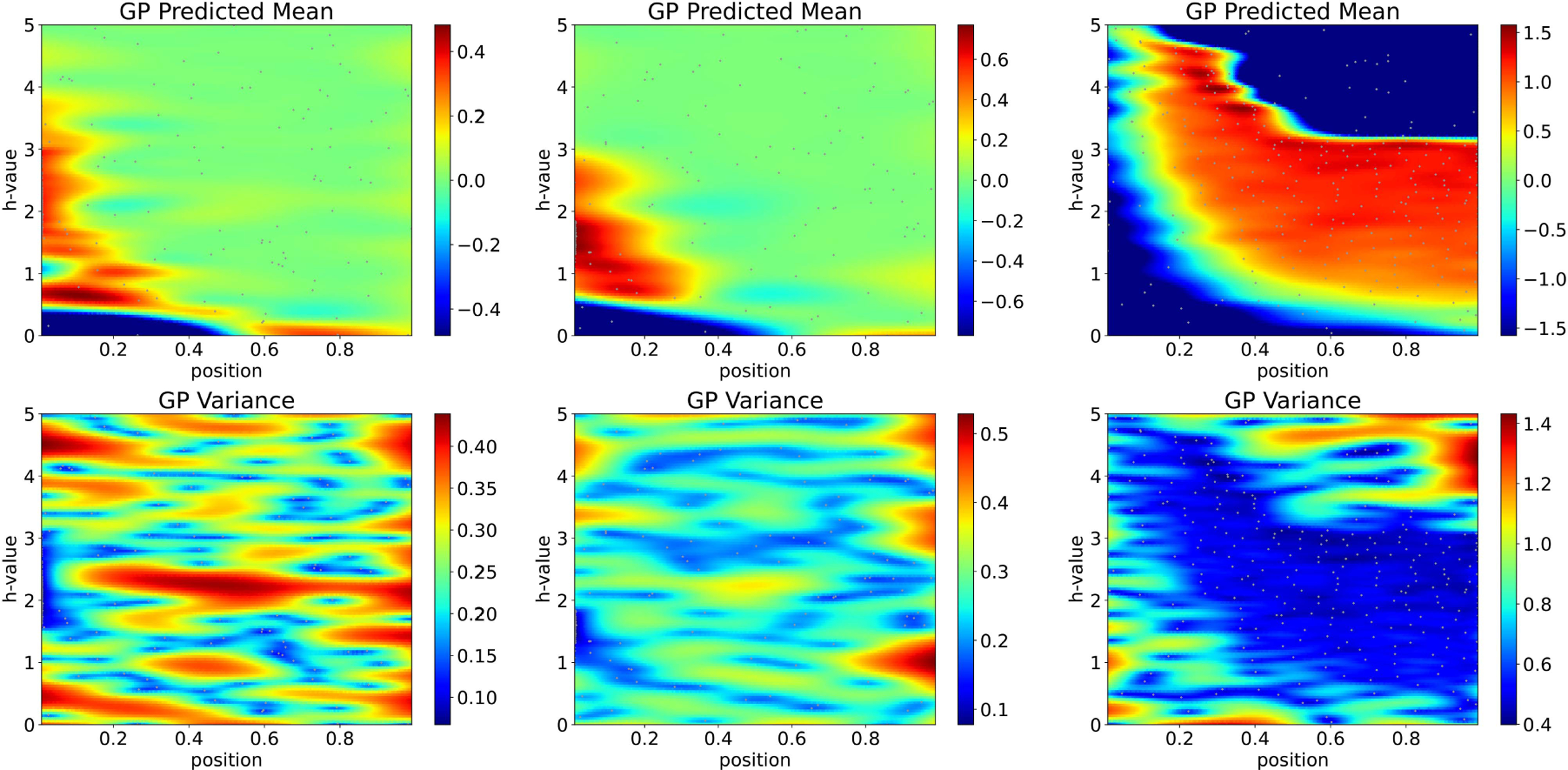
Bayesian optimization of the HG schedule for the weighted maximum clique problem on random graphs of (left column) density 0.1, (middle column) 0.5, and (right column) and 0.9. (Top subplots) Mean and (bottom subplots) variance of the Gaussian process (GP) used by the Bayesian optimizer, where the goal is to maximize the objective function (e.g., red regions mean that the constructed HG schedule resulted in better cut values being sampled). The parameters of the HG schedule for maximum cut are visualized as a heatmap for an annealing time of 1 *μ*s. Top plots show the maximum clique weight improvement as a function of g(t)∈[0,5] [see (3)] on the y-axis, where t is the position in the schedule (x-axis). The small gray points indicate the points where the objective function was evaluated. Each heatmap is computed from a total of 300 function calls (100 random points and 200 optimization steps). Data from DW_2000Q_LANL.

## APPENDIX A

### SCALING FACTORS FOR THE HG STATE ENCODING TECHNIQUE FOR MINOR EMBEDDED PROBLEMS

Table 2 shows the precise scaling factors $\alpha_{1}$ and $\alpha_{2}$ of (6) we employ to apply the HG technique of Section III-B, computed using the Bayesian optimization procedure. Those scaling factors depend on the density of the graph that each problem is applied to. The Ising formulation of the maximum cut problem of Section IV-B only requires one scaling factor, whereas the one of the maximum clique problem of Section IV-C requires two scaling factors.

## APPENDIX B

### CROPPED HARDWARE GRAPHS OF THE NATIVELY EMBEDDED SPIN GLASSES

The random Ising models embedded onto the D-Wave hardware for the QEMC experiments are chosen to match the entire native hardware graphs of each of the three QPUs used (see Table 1). An example of such hardware graphs is given in Fig. 11.

## APPENDIX C

### PARAMETER TUNING FOR THE WEIGHTED MAXIMUM CUT PROBLEM

The following two subsections elaborate on the tuning of the scaling factors, the annealing time, and the computation of the anneal schedules for the experiments involving the weighted maximum cut problem of Section IV-B.

#### A. SETTING SCALING FACTORS AND ANNEALING TIME

We start by determining a suitable choice of the scaling factor $\alpha_{1}$ in (6) for the maximum cut problem using the Bayesian optimization.

As a fitness function for the optimization, we use the improvement in the maximum cut over the baseline. Each time the optimizer issues a call to the fitness function, we supply the average of ten problems optimized with RA, HG, or RA+HG (depending on which one is optimized) using the parameter set probed by the Bayesian framework. The fitness value is then the average maximum cut improvement over the baseline. We make the fitness function dependent on three parameters, i.e., the scaling factor $\alpha_{1}$ as well as the parameters (h, t) determining the HG schedule (see Section III-E). For this experiment, the anneal time is set to 1 *μ*s.

After obtaining the fittest values, we fix the schedule (h, t) and the annealing time of 1 *μ*s and cross check the scaling factor $\alpha_{1}$ on a linear grid on [0.01,1] in increments of 0.01. Results for three different densities are shown in Fig. 12, which displays the difference in the maximum cut value to the baseline as a function of $\alpha_{1}$. We observe that the best choice of $\alpha_{1}$ is very dependent on the graph density, with, e.g., the best choice for density 0.9 occurring at $\alpha_{1}\approx 0.3$. Table 2 shows the precise optima we found for all nine densities p∈{0.1,…,0.9}. We will be selecting the scaling factor depending on the underlying graph density in the remainder of this section.

Next, we determine a suitable anneal duration for RA, HG, as well as the combined RA+HG. For this, we fix the HG schedule to the three points [0,5], [0.5T,2.5], [T,0] and the RA schedule to [0,1], [0.25T,0.25], [0.75T,0.25], [T,1], where T is the total annealing duration (given in Table 3). We note that these schedules are not optimal. Instead, they merely divide up the variable range in an equidistant fashion. We choose the anneal fraction to be around 0.25 since this is approximately where the crossover point of A(s) and B(s) is [66].

Table 3 shows maximum cut results for the smallest and largest possible anneal times as a function of the graph density. We observe that an annealing duration of 2000 *μ*s works best for RA, while a 1−μs anneal is best for HG. The combined technique of RA+HG does not seem to be affected by the annealing duration, but since an annealing time of 2000 *μ*s yields slightly better results, we decide to employ RA+HG in connection with a 2000−μs anneal in this section.

#### B. SCHEDULE COMPUTATION VIA BAYESIAN OPTIMIZATION

After having fixed the annealing duration for all three methods, we proceed by determining the parameters of the anneal schedule (see Section III-E) via Bayesian optimization. For each density, we carry out a single run of the Bayesian optimizer.

Since the schedule of HG has two parameters determining the midpoint in the anneal schedule (see Section III-E), we can visualize its optimization as a heatmap in Fig. 13. In particular, Fig. 13 shows the color-coded improvement in cut size over the baseline for each possible midpoint in the HG schedule. As described in Section III-E, this point consists of a position in the anneal and a value of the HG function g(t)∈[0,5] [see (3)]. Notice that the region of good HG schedules for the tested maximum cut problem instances can be directly read off of Fig. 13 (as opposed to the singular best schedules shown in Fig. 4), shown by the dark red region of the top subplot, where the y-axis h-value corresponds to the HG strength of the middle point of the HG schedule and the position on the x-axis corresponds to a proportion of the total annealing time.

The figure shows that the best choice of the HG value, defined as the one yielding the best improvement in maximum cut difference (red values), roughly decreases with the position in the anneal. We determine the maximum in this way for each density p∈{0.1,…,0.9}. The schedules for RA (three parameters) and RA+HG (five parameters) are fitted in a similar way, one schedule per density p∈{0.1,…,0.9}.

## APPENDIX D

### PARAMETER TUNING FOR THE MAXIMUM CLIQUE PROBLEM

The following two subsections elaborate on the tuning of the scaling factors, the annealing time, and the computation of the anneal schedules for the experiments involving the maximum clique problem of Section IV-C.

#### A. SETTING SCALING FACTORS AND ANNEALING TIME

We again focus first on the HG feature and repeat the tuning of Appendix C–A. In particular, to determine the two scaling factors $\alpha_{1}$ for h(x) and $\alpha_{2}$ for z, we run Bayesian optimization to fit both the schedule and the scaling factors simultaneously (see Section III-E). For this, we fix the anneal time at 1 *μ*s.

Each time the Bayesian optimizer requests a new point, we return the average maximum clique improvement overthe baseline (using 1000 anneals) for ten graphs for each density. If no solutions are found, i.e., z=−1 for all 1000 anneals, we return a large negative constant (we use −1000) to the optimizer.

After having obtained the result from the Bayesian optimization run, we fix the best schedule found. After initializing the Bayesian optimization algorithm with the parameters of the previous best solution (the previously found scaling constants $\alpha_{1}$ and $\alpha_{2}$ for the fixed schedule), we refit $\alpha_{1}$ and $\alpha_{2}$ with the help of the Bayesian optimization.

Fig. 14 shows the result of the Bayesian optimization run with a fixed annealing duration of 1 *μ*s and a graph density of p=0.5, as well as our fixed optimized schedule. We see that the best values for the scaling constants are essentially in a band around $\alpha_{1}=0.4$ (h-scale), with various maxima for $\alpha_{2}$ (z-scale). The precise optimal scaling factors for p=0.5 returned by the Bayesian optimization are $\alpha_{1}=0.35$ (for h-scale) and $\alpha_{2}=0.25$ (for z-scale), which we fix for the rest of this section.

We repeat this procedure for the other values of p∈{0.1,0.2,…,0.9} as well and use individual scaling constants for each density in the rest of this section as done for the maximum cut problem.

After having tuned the HG feature, we now focus again on the three techniques (RA, HG, and RA+HG). Similarly to Appendix C–A, we determine a suitable annealing duration for all three techniques by testing them for the shortest and longest annealing durations possible under the constraints of the Bayesian optimization we employed on Erdős–Rényi graphs of varying values of p∈{0,1,…,0.9}.

Results are displayed in Table 4, showing that RA works better for longer annealing times (especially for denser graphs), and HG works consistently better for shorter annealing durations. We will, thus, employ RA with an annealing time of 2000 *μ*s in the remainder of the minor embedded Bayesian optimization experiments, and HG with an annealing time of 1 *μ*s. For RA+HG, we fix the annealing duration at 2000 *μ*s.

#### B. SCHEDULE COMPUTATION VIA BAYESIAN OPTIMIZATION

The experiments of the previous sections allowed us to fix the anneal times, as well as the (density dependent) schedules and scaling constants for HG, RA, as well as RA+HG. Using the calibrations of the three methods, we now evaluate HG, RA, and RA+HG on graphs of varying density with respect to the improvement of the maximum clique weight over the baseline.

Similarly to Fig. 4(left), we also report the best schedules found by the Bayesian optimization in the case of the maximum clique problem. All schedules are optimized to maximize the maximum clique weight over the baseline in a standard FA.

Fig. 15 shows the resulting schedules. For RA, we see that, in contrast to the schedules for the maximum cut problem, for low densities, the optimal RA schedules decrease the anneal fraction at the start of the anneal to very small values and perform a pause until almost the full annealing time. As the graph under consideration becomes denser, the anneal fraction is only decreased down to roughly 0.4, and the pause occurs in the center having roughly a duration of half the annealing time. The HG schedules (see Fig. 15, right) resemble the ones observed for the maximum cut problem.

Similarly to Fig. 13, we again visualize the optimization of the HG schedule as a heatmap in Fig. 16. We see that the optimal point occurs at roughly position 0.2 (anneal fraction) and has an HG value of around 1. Notice that the region of good HG schedules for the tested maximum clique problem instances can be directly read off of Fig. 16 (as opposed to the singular best schedules shown in Fig. 15), shown by the red region of the top subplot, where the y-axis h-value corresponds to the HG strength of the middle point of the HG schedule and the position on the x-axis corresponds to a proportion of the total annealing time.

**TABLE 3.** Evaluation of RA and HG, as Well as RA+HG, for Smallest and Largest Possible Annealing Times (in Microseconds) Possible Under the Bayesian Optimization Methodology (Described in Section III-E)

	T [ms]	0.9	0.8	0.7	0.6	0.5	0.4	0.3	0.2	0.1
RA	100	0.722	−0.151	2.060	1.974	2.551	1.909	3.425	2.544	0.467
RA	2000	2.412	−0.149	3.653	2.140	3.261	2.859	4.507	3.209	0.610
HG	1	5.173	2.084	3.72	2.632	3.127	2.799	2.876	2.082	0.338
HG	2000	3.963	1.266	2.216	1.421	2.047	1.617	1.705	1.525	0.185
RA+HG	100	3.853	2.832	4.867	3.022	3.478	3.170	4.32	2.726	0.526
RA+HG	2000	4.145	2.540	5.001	2.725	4.222	3.128	4.526	3.113	0.566

Maximal cut difference on Erdos-Renyi graphs of density ranging from 0.1 to 0.9. Here, more positive values indicate that the solution planting method improved the mean cut-weight value found out of an average of the best solutions found from the ten fixed random graphs (per density). Data from DW_2000Q_LANL.

**TABLE 4.** Evaluation of RA and HG, as Well as Combined RA+HG, for Smallest and Largest Possible Anneal Times (in Microseconds) Allowed Using the Bayesian Optimization Method to Compute Good HG and Anneal Schedules (Described in Section III-E)

	anneal [ms]	0.9	0.8	0.7	0.6	0.5	0.4	0.3	0.2	0.1
RA	100	0.366	0.142	0.031	0.068	−0.309	−0.124	−0.391	−0.364	−0.322
RA	2000	0.481	0.289	−0.041	−0.060	−0.322	−0.171	−0.474	−0.408	−0.309
HG	1	1.195	1.307	1.263	0	0	0	0	0	0
HG	2000	0.908	1.004	1.018	0	0	0	0	0	0
RA+HG	100	0.780	−0.797	−3.874	0.104	0	0.659	0.442	0	0
RA+HG	2000	1.127	0.050	−2.167	0.011	0	0.610	0.309	0.039	0

Maximum Clique Difference on ErdOs-Renyi Graphs for Densities Ranging From 0.1 to 0.9.Here, more positive values indicate that the solution planting method improved the mean clique-weight value found out of an average of the best solutions found from the 10 fixed random graphs (per density).
